# Associations of *KCNQ1* Polymorphisms with the Risk of Type 2 Diabetes Mellitus: An Updated Meta-Analysis with Trial Sequential Analysis

**DOI:** 10.1155/2020/7145139

**Published:** 2020-07-03

**Authors:** Xiao-xuan Yu, Min-qi Liao, Yu-fei Zeng, Xu-ping Gao, Yan-hua Liu, Wei Sun, Sui Zhu, Fang-fang Zeng, Yan-bin Ye

**Affiliations:** ^1^Department of Epidemiology, School of Medicine, Jinan University, No. 601 Huangpu Road West, Guangzhou, 510632 Guangdong, China; ^2^Department of Obstetrics and Gynecology, Shangrao Fifth People's Hospital, Shangrao, Jiangxi 334000, China; ^3^The First Affiliated Hospital of Zhengzhou University, No. 1 East Jianshe Road, Zhengzhou, 450052 Henan, China; ^4^Customs Comprehensive Laboratory, Baiyun International Airport Customs, Hengyi Road, Guangzhou, 510080 Guangdong, China; ^5^Department of Medical Statistics, School of Medicine, Jinan University, No. 601 Huangpu Road West, Guangzhou, 510632 Guangdong, China; ^6^Department of Nutrition, The First Affiliated Hospital of Sun Yat-sen University, 58# Zhongshan Road 2, Guangzhou, 510080 Guangdong, China

## Abstract

**Background:**

Previous studies have examined the role of the KQT-like subfamily Q member1 (*KCNQ1*) gene polymorphisms on the risk of type 2 diabetes mellitus (T2DM), but the findings are inconclusive.

**Objective:**

To examine the association between the *KCNQ1* gene polymorphisms and the risk of T2DM using an updated meta-analysis with an almost tripled number of studies.

**Methods:**

Five electronic databases, such as PubMed and Embase, were searched thoroughly for relevant studies on the associations between seven most studied *KCNQ1* gene polymorphisms, including rs2237892, rs2237897, rs2237895, rs2283228, rs231362, rs151290, and rs2074196, and T2DM risk up to September 14, 2019. The summary odds ratios (ORs) with their 95% confidence intervals (CIs) were applied to assess the strength of associations in the random-effects models. We used the trial sequential analysis (TSA) to measure the robustness of the evidence.

**Results:**

49 publications including 55 case-control studies (68,378 cases and 66,673 controls) were finally enrolled. In overall analyses, generally, increased T2DM risk was detected for rs2237892, rs2237895, rs2283228, rs151290, and rs2074196, but not for rs231362 under all genetic models. The ORs and 95% CIs for allelic comparison were 1.23 (1.14-1.33) for rs2237892, 1.21 (1.16-1.27) for rs2237895, 1.27 (1.11-1.46) for rs2237897, 1.25 (1.09-1.42) for rs2283228, 1.14 (1.03-1.27) for rs151290, 1.31 (1.23-1.39) for rs2074196, and 1.16 (0.83, 1.61) for rs231362. Stratified analyses showed that associations for rs2237892, rs2237895, rs2283228, and rs151290 were more evident among Asians than Caucasians. TSA demonstrated that the evidence was sufficient for all polymorphisms in this study. The genotypes of the three SNPs (rs2237892, rs2283228, and rs231362) were significantly correlated with altered KCNQ1 gene expression.

**Conclusion:**

This meta-analysis suggested that *KCNQ1* gene polymorphisms (rs2237892, rs2283228, rs2237895, rs151290, and rs2074196) might be the susceptible factors for T2DM, especially among Asian population.

## 1. Introduction

The global epidemic of diabetes is a major public health problem, and the number of cases has increased four times in the past 30 years [1]. Incredibly, 1 in 11 adults suffered diabetes globally. It is estimated that about 463 million adults were living with diabetes mellitus worldwide in 2019, and most of them had type 2 diabetes mellitus (T2DM). Moreover, this number is expected to increase to 642 million by 2040 [2]. Considering its high prevalence and rapid increasing speed, there are increasing numbers of investigations focusing on risk factors and susceptibilities for T2DM. However, the underlying etiology of T2DM remains unclear. Therefore, we conducted this meta-analysis to further demonstrate whether genetic factors play a vital role in the pathogenesis of T2DM [3] or not.

KQT-like subfamily Q member1 (*KCNQ1*) is a family of voltage-gated potassium channels, and the *KCNQ1* gene located on 11p15.5 encoded the protein [4]. Although KCNQ1 is mainly expressed in the tissues or cells in the heart, it is also expressed in other tissues or organs such as pancreas islets [5]. Blockading the channels with *KCNQ1* inhibitors, 293B might stimulate secretion of insulin in pancreas, suggesting the effect of *KCNQ1* on the regulation of insulin secretion [6].

Unoki et al. [7], firstly, noted that populations with *KCNQ1* gene polymorphisms were susceptible to T2DM. Since then, many studies have explored the roles of *KCNQ1* gene polymorphisms on T2DM risk, but their findings were inconsistent. These discrepancies might be attributable to the various types of research populations and different sample sizes across studies. The statistical testing power of a single study may not be enough to detect the small effect. Therefore, it is necessary to conduct a comprehensive analysis on the association between the *KCNQ1* polymorphisms and the risk of T2DM. Therefore, in 2013, a meta-analysis of Liu et al. [8] consequently suggested that *KCNQ1* rs2237892, rs2237897, rs2237895, rs2283228, and rs231362 polymorphisms were associated with increased T2DM risk. Nevertheless, this meta-analysis only paid attention to the risk of alleles for type 2 diabetes, lacking evidence to clarify the risk for T2DM in different genetic models. Furthermore, 31 additional related papers [9–39] have been published after the publication of this meta-analysis; hence, the sample size became three times more.

Thus, this updated meta-analysis with trial sequential analysis (TSA) of all eligible studies was performed to assess the effect of the *KCNQ1* polymorphisms on T2DM risk. With 49 eligible articles included, we aimed to assess the associations between the 7 most studied polymorphisms of *KCNQ1* (rs2237892, rs2237895, rs2237897, rs2283228, rs231362, rs151290, and rs2074196) and the T2DM risk.

## 2. Methods and Analysis

### 2.1. Study Identification

Five electronic databases (PubMed, Embase, Web of Science, China National Knowledge Infrastructure (CNKI), and Wanfang databases) were systematically searched up to July 3, 2019, using combinations of the following keywords: Diabetes Mellitus[MESH]/diabetes and *KCNQ1* and Polymorphism[MESH]/polymorphism/variant/genotype without language restriction. Some references might be inevitably missing during the database searching. Therefore, the corresponding items generated by PubMed have been recovered. The reference lists of the main related studies and review studies were reviewed to identify potential missing articles which are related to this topic.

### 2.2. Selection Criteria

Two authors (X-XY and M-QL) independently reviewed and selected the studies. The following inclusion criteria were used to select studies for this meta-analysis: (1) case-control study as study design; (2) T2DM as outcome; (3) evaluation of at least one of the following seven polymorphisms: rs2237892, rs2237895, rs2237897, rs2283228, rs231362, rs151290, and rs2074196; and (4) enough genotype information for data analysis. Studies with gestational diabetes or postoperative diabetes as the endpoint were excluded. Only the studies with the largest sample size were included when there were duplicate studies with overlapping data.

### 2.3. Data Extraction

For the included studies, the data were abstracted by two authors (XY and ML) using the same data extraction form as follows: first author, year of publication, country of origin or ethnicity of study participants, source of control groups, sample size of cases and controls, mean age and percentage of male of cases and controls, genotyping method, T2DM diagnostic criteria used, matching variables, and genotype and allele distributions of cases and controls.

### 2.4. Quality Assessment

The method, derived from a meta-analysis by Thakkinstian et al. [40] in 2011, was used to assess the methodological quality of the included case-control studies. The quality assessment criteria are shown in Supplementary Table 1. The quality for each included study based on their criteria was evaluated and given a score from 0 to 15. If a study was given a score of 9 or above, it was classified as a high-quality study. The same two authors mentioned above scored each study separately and independently, and the controversy about each study was discussed to reach a consensus.

### 2.5. Quantitative Synthesis

The chi-squared goodness-of-fit test was used to assess whether the gene frequency in controls was consistent with the Hardy-Weinberg equilibrium (HWE). The associations of seven polymorphisms of *KCNQ1* (rs2237892, rs2237895, rs2237897, rs2283228, rs231362, rs151290, and rs2074196) with the T2DM risk were evaluated by the collected odds ratios (ORs) and their 95% confidence intervals (95% CIs) according to the following five genetic models: (1) allele genetic model: M vs. W (“W” is the wild allele and “M” is the mutant allele); (2) homozygote genetic model: MM vs. WW; (3) heterozygote genetic model: WM vs. WW; (4) dominant genetic model: MM+WM vs. WW; and (5) recessive genetic model (MM vs. WM+WW). Heterogeneity between studies was assessed using Cochran's*Q*and the*I*^2^statistics. The random-effects model by the DerSimonian and Laird method was used in all analyses regardless of *I*^2^ values because it provides more conservative estimates [41].

The stratified analyses and metaregression analyses were performed by races (Asian, Caucasian, or Mixed), HWE (yes or no), control sources (population or hospital), quality scores (<9 or ≥9 points), year of publication (<2015 or ≥2015), and number of participants (<500 or ≥500), respectively. Sensitivity analysis by sequentially removing each study was also applied to verify the robustness of our findings [42]. The published bias was tested by Egger's and Begg's regression asymmetry, as well as the funnel plots [43, 44].

All analyses mentioned above were conducted using Stata 11.0 software, and the test level was a *P* < 0.05 on two sides.

### 2.6. Trial Sequential Analysis

The TSA tool was used to evaluate whether the quantitative results are reliable, and the required information size (RIS) was calculated to reduce type I error [45, 46]. TSA was performed for T2DM by anticipating a 20% relative risk reduction, a 5% of type I error, and an 80% of statistical test power [45].

### 2.7. Genotype-Based mRNA Expression Analysis

The *KCNQ1* mRNA expression data was retrieved for *KCNQ1* SNPs from the GTEx Portal database (https://www.gtexportal.org/home/). These data were used to evaluate the correlation between SNP genotypes and *KCNQ1* mRNA expression level alteration.

## 3. Results

### 3.1. Study Selection and Characteristics

**Figure 1:**
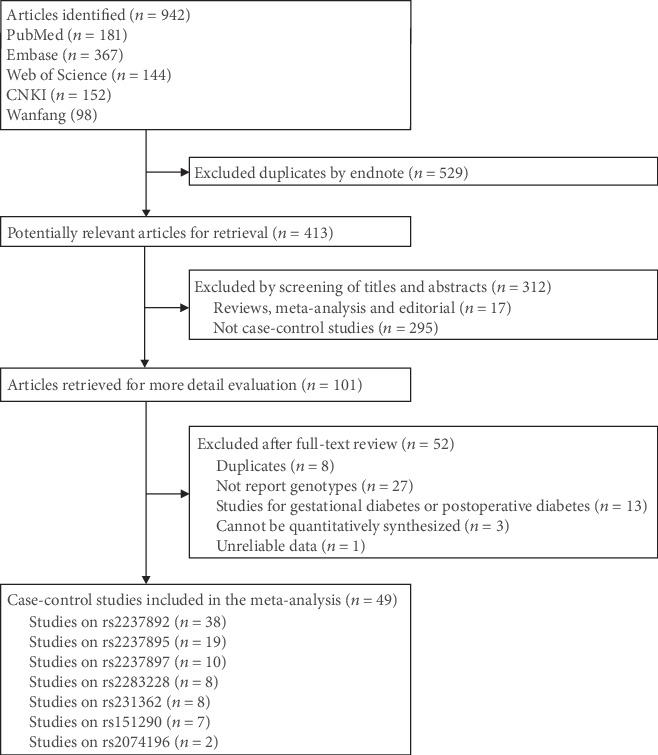
Flow diagram of the literature search and selection process.

In total, 942 articles were identified through the literature search (Figure 1). After the removal of duplicated articles and articles that did not meet the inclusion criteria, ultimately, 49 articles [7, 9–39, 47–63] (68,378 T2DM cases and 66,673 controls) were included in the qualitative synthesis.

The main characteristics for each included study are presented in Table 1, including studies from 15 different countries published from 2008 to 2018. The sample sizes ranged from 60 to 15,577 (median 900). There are 42,096 population-based controls and 24,577 hospital-based controls. The mean ages studied were 50.6 and 51.6 years for cases and controls, respectively. The proportions of male were 51.2% in the case group and 45.4% in the control group. Controls in 29 (52.7%) studies were matched at least by one variable (e.g., age, gender). Supplementary Table 2 shows the genotype distributions and HWE status of each polymorphism of all included studies.

Forty-two studies (50,747 cases and 50,023 controls) were included in the meta-analysis for rs2237892, 23 studies (42,127 cases and 38,276 controls) for rs2237895, 12 studies (18,808 cases and 18,847 controls) for rs2237897, and 10 studies (13,188 cases and 12,191 controls) for rs2283228. Besides, there are less than ten studies focused on rs231362, rs151290, and rs2074196, respectively.

### 3.2. Quantitative Synthesis

The summary of pooled estimates for the association between *KCNQ1* polymorphisms and T2DM risk is provided in Table 2 and Supplementary Fig. 1. Overall, the increased T2DM risks were generally found for rs2237892, rs2237895, rs2237897, rs2283228, rs151290, and rs2074196 under allele comparison, as well as all genetic models. For allele comparison, the ORs and their 95% CIs were 1.23 (1.14, 1.33) for the allele C of rs2237892, 1.21 (1.16, 1.27) for the allele C of rs2237895, 1.27 (1.11, 1.46) for the allele C of rs2237897, 1.25 (1.09, 1.42) for the allele A of rs2283228, 1.14 (1.03, 1.27) for the allele C of rs151290, and 1.31 (1.23, 1.39) for the allele G of rs2074196, respectively. On the contrary, no association was found for rs231362 G/A polymorphism under any genetic model (OR: 1.16-1.51; *P* ranges: 0.12-0.39). Moderate to significant heterogeneity was observed for most of these polymorphisms (e.g., *I*^2^ for allelic comparison ranged from 39.7% to 94.7%).

The results of subgroup analyses were revealed in Supplementary Table 3. When stratified by race, with studies conducted only among Asians for rs2237897, the risk effect was more evident among Asians than among Caucasians for rs2237892, rs2237895, rs2283228, and rs151290. All the risks persisted after excluding studies that deviated from HWE. When stratified by the sources of controls, the significant risk effects were only noticed in a population-based subgroup for rs2237892 and rs151290, whereas similar associations were observed for rs2237895 and rs2237897. In the subgroup analyses by quality scores and sample size, in general, the statistically significant results tended to occur in studies with high quality score and larger sample size. For rs2237892, rs2237895, and rs151290, the interaction of race on the association between *KCNQ1* polymorphisms and T2DM has been demonstrated by metaregression analyses (*P* for regression: 0.041, 0.008, and 0.057, respectively; Supplementary Table 4).

### 3.3. Sensitivity Analysis

The sensitivity analysis indicated that most of our results in all genetic models for the 7 included polymorphisms were robust after excluding any single study, except for the results for the heterozygote comparison of rs2237897, the allelic comparison and recessive genetic model of rs231362, and the allelic comparison and recessive genetic model of rs151290, after excluding the study by Wang et al. [17], Ohshige et al. [59], and Yasuda et al. [48], respectively.

### 3.4. Publication Bias

Possible publication bias was assessed by Begg's and Egger's test, as well as the funnel plots. Begg's funnel plots of seven polymorphisms were basically symmetrical, and all *P* values for Egger' test were greater than 0.05. Our results indicated that publication bias does not appear in any comparison (Supplementary Fig. 2 and Supplementary Table 5).

### 3.5. Trial Sequential Analysis Results

The TSA was performed to investigate the relevance of *KCNQ1* seven gene polymorphisms with T2DM susceptibility. The allelic comparison was used to study all these polymorphisms. We noticed that the total number of cases and controls for all current relevant studies has exceeded the amount of information required for rs2237892, rs2237895, rs2283228, rs151290, and rs2074196 but insufficient for rs2237897 or rs231362 (Supplementary Fig. 3). Although the RIS has not yet been reached, the cumulative *Z*-curve crossed the monitoring boundary for rs2237897. Thus, the effect of rs2237897 on T2DM risk was stable. For rs231362, the RIS of 29011 has not yet been reached, but the limit of futility has been reached.

### 3.6. Genotype-Based *KCNQ1* mRNA Expression Analysis Results

Through the GTEx Portal website, mRNA expression data of three genotypes of rs2237892, rs2283228, and rs231362 were obtained. We found that the genotypes of the three SNPs were significantly correlated with altered *KCNQ1* gene expression (rs2237892 C/T: *P* = 3.8∗10^−5^; rs2283228 A/C: *P* = 6.3∗10^−5^; and rs231362 G/A: *P* = 3.5∗10^−5^) (Supplementary Figs. 4–6).

## 4. Discussion

To our knowledge, this meta-analysis is the most comprehensive study of the association between the *KCNQ1* seven gene polymorphisms and the T2DM risk. Our results provide evidence that six *KCNQ1* polymorphisms (rs2237892, rs2237897, rs151290, rs2283228, rs2074196, and rs2237895) might be significantly associated with increased T2DM risk. These significant associations are more pronounced among Asian populations and can be further confirmed by TSA.

Two previous meta-analyses [8, 64] focusing on the association between the *KCNQ1* polymorphisms and the risk of T2DM were published in 2013 and 2014, respectively. However, only the allele genetic model was analyzed in the meta-analysis by Liu et al. [8] and only one SNP of *KCNQ1* was studied in the meta-analysis by Li et al. [64]. But, seven SNPs of *KCNQ1* according to not only the allele genetic model but also the genetic models of the homozygote, heterozygote, dominant, and recessive comparison were performed in this meta-analysis. In the meta-analysis by Liu et al. [8], significantly increased T2DM risks were found for C allele of rs2237892 (OR = 1.31; *P* < 0.001), C allele of rs2237895 (OR = 1.24; *P* < 0.001), C allele of rs2237897 (OR = 1.34; *P* < 0.001), A allele of rs2283228 (OR = 1.23; *P* < 0.001), and G allele of rs231362 (OR = 1.10; *P* < 0.001). Compared to the study by Liu et al. [8], 31 new articles [9–39] have been added to this meta-analysis, and the significant risks for rs151290 and rs2074196 were firstly found by us. Our results confirmed the effects of rs2237892, rs2237895, rs2237897, and rs2283228 on T2DM risk, but the result for rs231362 contradicted previous studies [8]. The following reasons may explain this discrepancy. First, the meta-analysis by Liu et al. [8] used a random-effects model only when there was heterogeneity; otherwise, a fixed-effects model was used. In this study, we always use the random-effects model, so our results are conservative. But for rs231362, with actual heterogeneity, that is, a random-effects model should be used. Second, Liu et al. [8] only studied the allele comparisons of *KCNQ1* polymorphisms (rs2237892, rs2237895, rs2237897, rs2283228, and rs231362) and did not conduct research on genotype distribution. Therefore, correspondingly, its literature selection criteria are more lenient. The inclusion of the literature not included in this article may lead to statistically significant results for the rs231362 allele. Finally, the results of the study by Liu et al. [8] of Asians with rs231362 are consistent with this article—there is no significant association; significant association only occurred among Caucasians. For rs231362, the meta-analysis of Liu et al. [8] included only one study of Caucasians, which was a large-sample study. Therefore, we suspect that it caused the bias of the results of the overall population in the meta-analysis of Liu et al. [8]. There was no statistically significant association in the Caucasian subgroup in this meta-analysis, most likely because our relatively strict selection criteria—enough genotype information was required—did not allow the study to be included. Another study by Li et al. [64], with 9 studies included (6707 cases and 8129 controls), was only performed for rs2237892. They found a significant association between *KCNQ1* rs2237892 T polymorphism and T2DM in the Asian population under the allelic (OR = 1.350; *P* < 0.001), recessive (OR = 0.650; *P* < 0.001), dominant (OR = 1.450; *P* < 0.001), and additive genetic models (OR = 1.346; *P* < 0.001). The result of recessive genetic models contradicted with this meta-analysis (OR = 1.39; *P* < 0.001). Similarly, compared to the study by Li et al. [64], 28 new articles [10, 13, 15–22, 24, 26, 27, 30–39, 48, 52, 60, 62, 63] have been added into the present meta-analysis; therefore, the existence of the inconsistent results between their study and ours is reasonable.

There is biological evidence supporting the hypothesis that *KCNQ1* might play a role in the susceptibility of T2DM. *KCNQ1*, encoding the alpha subunit of the IKsK^+^ channel, is mainly expressed in the tissues or cells of the heart [65], as well as in pancreas islets, which plays an important role in the regulation of insulin secretion [7, 66]. The variants of *KCNQ1* are associated with impaired fasting glucose, beta-cell function, and impaired metabolic traits [7, 48]. Studies in INS-1 cells indicated that *KCNQ1*, assembling with KCNE2 in insulin-secreting cells, could block the *KCNQ1* K^+^ channel with the sulfonamide analogue 293B and reduce 60% of whole beta-cell outward currents and that the presence of both 293B and tolbutamide could significantly increase the insulin secretion [6]. Animal studies revealed that increased *KCNQ1* protein expression could limit insulin secretion in pancreatic beta-cells through regulating potassium channel currents [67]. The previous study in vitro indicated that risk SNPs increase *KCNQ1* expression in pancreatic beta-cells, which increased the risk of T2DM [68]. And the research by Zeng et al. [69] showed that mutation of *KCNQ1* impaired capacity to maintain glucose homeostasis in vivo. A previous population-based study has indicated that the three polymorphisms of *KCNQ1* (rs2237892, rs2237895, and rs2237897) were significantly associated with the OGTT-derived insulin secretion index [70]. In addition, the rs151290 gene polymorphism was significantly related with the 30-minute C-peptide level during OGTT, the first-stage insulin secretion, and the proinsulin index [70]. It was also suggested that the methylation difference of *KCNQ1* was associated with insulin sensitivity and that CpG site-specific genetic variation predicted the methylation difference [71]. The molecular mechanism by which *KCNQ1* is associated with the risk of T2DM may be explained by the reasons mentioned above. We also found that under different genotypes of rs2237892, rs2283228, or rs231362, the expression level of *KCNQ1* gene was significantly different. However, more research studies should be conducted to test the association between other SNPs with *KCNQ1* gene expression levels.

After stratified by race, the estimated risks were more evident among Asians than among Caucasians for rs2237892, rs2237895, rs2283228, and rs151290. Metaregression analyses also confirmed these phenomena. According to our data, the significantly lower proportion in the frequency of minor alleles in controls for *KCNQ1* polymorphisms was observed among Asians and Caucasians (e.g., in rs2237892, 5.6% vs. 34.6%; in rs2283228, 6.5% vs. 37.5%; and in rs151290, 25.1% vs. 41.8%), which might have led to a difference in the results between the two ethnicities.

Since 2008, many related original studies or meta-analyses on the association of *KCNQ1* and T2DM have been published. However, none of these meta-analyses has conducted the TSA, suggesting the lack of support from TSA. Accompanied with our results, the TSA in our study showed that the RIS had been achieved in each SNP. Thus, the findings of the present meta-analysis are suggested to be robust.

Following the PRISMA guidelines, our meta-analysis was carried out through conducting a comprehensive literature search, using Egger's and Begg's regression asymmetry test and funnel plot to assess potential publication bias, and exploring the potential sources of heterogeneity by subgroup and sensitivity analyses. Our findings persisted after excluding studies that deviated from HWE. This meta-analysis had included the largest number of existing original studies in this area; therefore, the statistical testing power was relatively higher than before. In addition, a TSA method has been used to test the robustness of our findings, which might further ensure our results.

The findings of the present study should be mentioned with some limitations. Firstly, we noted the heterogeneity in the overall effect estimated for most of the SNPs which have been quantitatively synthesized. However, we had tried to explore the potential sources of heterogeneity by subgroup or metaregression analyses, and we found that ethnicity might be the main potential source of between-study heterogeneity in rs2237892, rs2237895, rs2283228, and rs151290. Secondly, in the sensitivity analysis, we found that the results of two genetic models for rs231362 would reach significance after removing the study by Ohshige et al. [59]. Nevertheless, the sample size of this study by Ohshige et al. [59] is the largest for rs231362, indicating that this result was needed to be further confirmed. Thirdly, the number of pooled studies for the subgroup analyses was relatively small for rs2074196 (*n* = 4), and it might have attenuated the statistical power and confined the conduction of subgroup analyses. Fourthly, all the included studies for rs2237897 are derived from Asians, and more studies with diverse race are needed to confirm its role on T2DM risk. Finally, due to the lack of information about the age of onset of a large number of studies, we cannot assess the effect of the *KCNQ1* polymorphism on the T2DM according to the age at baseline, which may also affect the further interpretation of our study.

In conclusion, our results demonstrate that the C allele of rs2237892, rs2237895, and rs151290, the A allele of rs2283228, and the G allele of rs2074196, but not C allele of rs231362 of *KCNQ1* gene, might play significant roles in the susceptibility of T2DM, especially among Asian population. Still, larger and well-designed studies including other risk factors are warranted to validate the findings from the present analysis.

## Figures and Tables

**Table 1:** Study characteristics from included studies in the meta-analysis.

First author	Year	Country	Ethnicity	Cases			Controls			Control source	Matching variables	SNP(s)^d^	Score
				*N* ^a^	Age (y)^b^	Gender^c^	*N* ^a^	Age (y)^b^	Gender^c^				
Lee YH	2008	Korea	Asian	908	58.2 ± 11.1	48.4	502	55.0 ± 9.4	53.6	Hospital	—	a	9
Unoki H (Japanese)	2008	Japan	Asian	5149	—	61.4	4176	—	47.6	Hospital	—	b, c, d	9
Unoki H (Chinese)	2008	China	Asian	1498	63.9 ± 9.7	49.1	1881	35.4 ± 11.2	44.0	Hospital	—	b, c, d	8
Unoki H (Danish)	2008	Denmark	Caucasian	4085	60.0 ± 9.8	59.3	5032	46.9 ± 9.1	53.7	Hospital	—	b, c, d	9
Yasuda K (Japanese)	2008	Japan	Asian	4378	—	—	4412	—	—	Population	—	a, b, f	12
Yasuda K (Chinese)	2008	China	Asian	1416	50.0 ± 13.7	40.4	1577	25.1 ± 14.2	46.1	Population	—	a, b	11
Yasuda K (Korean)	2008	Korea	Asian	758	59.2 ± 9.9	46.7	632	64.7 ± 3.6	45.4	Population	Gender	a, b	8
Yasuda K (Caucasian)	2008	Sweden	Caucasian	2830	57.9 ± 11.5	58.9	3740	57.4 ± 6.0	37.9	Population	Age	a, b	10
Hu C	2009	China	Asian	1769	61.1 ± 12.6	52.1	1734	57.4 ± 12.4	41.4	Hospital	—	a, b, c	7
Liu Y	2009	China	Asian	1912	63.9 ± 9.5	41.1	2041	58.1 ± 9.4	31.1	Population	—	a, b, c	10
Chen Z	2010	China	Asian	57	—	—	341	—	—	Hospital	—	a, b, c, d	6
Dehwah MAS	2010	China	Asian	223	53.9 ± 10.3	44.8	201	66.5 ± 8.0	44.3	Hospital	Gender	a	7
Han X	2010	China	Asian	1024	56.0 ± 12.0	52.7	1005	58.0 ± 9.0	34.1	Population	Age	a	11
Xu M	2010	China	Asian	66	—	—	652	—	—	Population	—	a	9
Wei Q	2010	China	Asian	133	56.0 ± 8.5	51.1	106	57.7 ± 11.1	50.9	Hospital	Age and gender	c, d, f	6
Zhang L	2010	China	Asian	100	53.0 ± 12.0	58.0	97	47.0 ± 18.0	53.6	Population	—	b	8
Been LF	2011	India and the USA	Caucasian	1428	—	—	1593	—	—	Population	—	a, b, e	13
Ohshige T	2011	Japan	Asian	2839	62.8	50.0	2125	51.6	50.0	Hospital	Gender	e	9
Saif-Ali R (Chinese)	2011	Malaysia	Asian	300	49.8 ± 7.4	51.0	230	52.9 ± 9.2	61.3	Hospital	Age	a, b, d	8
Saif-Ali R (Malay)	2011	Malaysia	Asian	234	48.5 ± 7.5	45.3	117	44.9 ± 10.7	45.8	Hospital	Age and gender	a, b, d	7
Tabara Y	2011	Japan	Asian	506	—	—	402	—	—	Hospital	—	a	8
Shi L	2011	China	Asian	171	56.1 ± 12.9	56.1	288	48.9 ± 11.6	60.1	Hospital	—	c, f	3
Van JV	2012	Netherlands	Caucasian	4620	64.3 ± 10.6	43.3	5285	51.1 ± 10.1	41.9	Population	Gender	a, b, f	12
Dai XP	2012	China	Asian	367	49.1 ± 10.8	49.9	214	47.6 ± 10.9	57.9	Hospital	Age	a, b	8
Iwata M	2012	Japan	Asian	724	64.9 ± 11.1	62.3	763	72.5 ± 9.0	47.1	Hospital	—	a, e	9
Lu S	2012	China	Asian	498	56.0 ± 7.0	59.6	402	49.0 ± 9.01	66.4	Hospital	—	e	8
Turki A	2012	Tunisia	Caucasian	900	61.2 ± 9.7	37.8	600	52.0 ± 11.9	45.5	Hospital	—	a, b, d, f	8
Gao X	2012	China	Asian	200	55.4 ± 12.8	55.5	200	53.7 ± 8.8	49.5	Hospital	Age	f	8
Wang J	2012	China	Asian	300	54.9 ± 13.2	42.3	100	52.6 ± 11.9	42.0	Hospital	Age and gender	e	7
Almawi WY	2013	Lebanon	Caucasian	995	58.6 ± 13.4	58.8	1076	57.3 ± 10.4	47.0	Hospital	Age	a, b	8
Wang H	2013	China	Asian	2533	53.3	59.6	2643	56.1	55.3	Population	Age and gender	a, b, c, d	10
Lin YD	2013	China	Asian	2925	58.2 ± 10.1	37.5	3281	56.6 ± 9.9	37.6	Population	Age and gender	a, b, c, e	11
Yang HL	2013	China	Asian	222	52.6 ± 20.5	55.9	140	59.4 ± 15.2	43.6	Hospital	—	a	6
Yu WH	2013	China	Asian	9221	58.1	48.5	4052	48.6	40.7	Population	—	a, b	11
Bazzi MD	2014	Saudi Arabia	Caucasian	90	50.7 ± 11.7	50.0	95	40.6 ± 4.6	52.6	Hospital	Age and gender	a	7
Sun ZH	2014	China	Asian	321	—	—	345	—	—	Hospital	—	c	8
Zhang LW	2014	China	Asian	349	49.5 ± 8.1	55.9	300	48.8 ± 11.7	60.3	Population	Age	a, c	11
Zhu AN	2014	China	Asian	238	58.3 ± 11.9	55.9	240	57.7 ± 11.6	55.8	Hospital	Age and gender	a	7
Khan IA	2015	India	Mixed	250	57.2 ± 8.2	55.2	250	53.9 ± 6.3	57.6	Hospital	Age and gender	d	7
Zhang W	2015	China	Asian	530	61.0 ± 12.6	53.0	452	58.8 ± 11.4	50.9	Hospital	Age and gender	a	8
Guo HL	2015	China	Asian	30	46.7 ± 7.3	63.3	30	46.6 ± 12.6	43.3	Hospital	Age	a	5
Shen Q	2015	China	Asian	922	58.5 ± 12.3	51.5	925	50.0 ± 7.5	46.4	Hospital	—	a	9
InterAct Consortium	2016	European countries	Mixed	6869	—	—	8708	—	—	Population	—	a	4
Cui LJ	2016	China	Asian	100	51.2 ± 11.6	55.0	100	49.9 ± 12.4	46.0	Hospital	Age	a, b	6
Gao K	2016	China	Asian	736	52.5 (43-61)	57.9	768	47.0 (39.0-57.0)	42.2	Population	—	f	11
Riobello C	2016	Spain	Caucasian	180	—	—	501	—	—	Population	—	a, b, e	11
Zhou X	2016	China	Asian	305	50.1 ± 6.4	49.5	200	48.9 ± 11.9	50.1	Hospital	Age and gender	a	8
Al-Shammari MS	2017	Saudi Arabia	Caucasian	320	51.5 ± 8.8	54.4	516	48.8 ± 6.9	56.0	Hospital	Age and gender	a, b, f	6
Baniasadian S	2018	Iran	Caucasian	77	—	45.5	90	—	48.9	Hospital	Gender	a	3
Plengvidhya N	2018	Thailand	Asian	500	57.2 ± 12.2	67.2	500	53.0 ± 8.4	71.2	Hospital	—	a	8
Chen JF	2018	China	Asian	84	54.5 ± 13.5	59.5	104	51.5 ± 11.2	55.8	Hospital	Age	a	6
Huang Q	2018	China	Asian	513	55.3 ± 6.6	28.5	502	55.2 ± 6.7	29.9	Population	Age and gender	a, f	13
Li YH (Uighur)	2018	China (Uighur)	Asian	282	48.3 ± 10.6	48.2	99	—	49.5	Population	Gender	a, e	8
Li YH (Chinese Han)	2018	China (Chinese Han)	Asian	293	59.4 ± 13.2	56.0	208	—	47.1	Population	—	a, e	10
Xu T	2018	China	Asian	100	50.6 ± 7.5	68.0	100	48.9 ± 8.4	57.0	Hospital	Age	a	5

SNP: single nucleotide polymorphism. ^a^Number, ^b^age at survey, ^c^percentage of male, ^d^SNPs: a: rs2237892; b: rs2237895; c: rs2237897; d: rs2283228; e: rs231362; f: rs151290.

**Table 2:** Total analysis of seven *KCNQ1* gene polymorphisms on T2DM risk.

Variables	*N* ^a^	Cases/controls	Allelic comparison				Homozygote comparison				Heterozygote comparison				Dominant genetic model				Recessive genetic model			
			OR (95% CI)	*P* ^b^	*I* ^2^ (%)	*P* ^c^	OR (95% CI)	*P* ^b^	*I* ^2^ (%)	*P* ^c^	OR (95% CI)	*P* ^b^	*I* ^2^ (%)	*P* ^c^	OR (95% CI)	*P* ^b^	*I* ^2^ (%)	*P* ^c^	OR (95% CI)	*P* ^b^	*I* ^2^ (%)	*P* ^c^
rs2237892	42	50,747/50,023	1.23(1.14, 1.33)	<0.01	88.7	<0.01	1.69(1.45, 1.96)	<0.01	80.2	<0.01	1.40(1.28, 1.54)	<0.01	44.3	<0.01	1.55(1.37, 1.76)	<0.01	71.9	<0.01	1.24(1.12, 1.36)	<0.01	87.7	<0.01
rs2237895	23	42,127/38,276	1.21(1.16, 1.27)	<0.01	74.1	<0.01	1.45(1.32, 1.60)	<0.01	70.7	<0.01	1.23(1.17, 1.29)	<0.01	48.6	0.01	1.28(1.21, 1.36)	<0.01	65.9	<0.01	1.31(1.22, 1.41)	<0.01	56.9	<0.01
rs2237897	12	18,808/18,847	1.27(1.11, 1.46)	<0.01	92.5	<0.01	1.49(1.09, 2.03)	0.01	91.6	<0.01	1.19(0.96, 1.48)	0.11	84.2	<0.01	1.34(1.02, 1.74)	0.03	91.2	<0.01	1.36(1.16, 1.60)	<0.01	88.7	<0.01
rs2283228	10	13,188/12,191	1.25(1.09, 1.42)	<0.01	81.8	<0.01	1.53(1.25, 1.87)	<0.01	50.3	0.03	1.20(1.08, 1.33)	<0.01	0.0	0.90	1.33(1.18, 1.49)	<0.01	10.9	0.34	1.30(1.10, 1.55)	<0.01	83.2	<0.01
rs231362	9	7666/6626	1.16(0.83, 1.61)	0.39	94.7	<0.01	1.51(0.90, 2.53)	0.12	77.1	<0.01	1.22(0.94, 1.58)	0.14	24.5	0.23	1.33(0.90, 1.97)	0.15	65.1	<0.01	1.22(0.82, 1.8)	0.33	94.6	<0.01
rs151290	9	7808/7836	1.14(1.03, 1.27)	0.02	71.2	<0.01	1.35(1.09, 1.66)	<0.01	61.6	0.01	1.20(1.04, 1.38)	0.01	27.5	0.20	1.26(1.08, 1.47)	<0.01	41.1	0.09	1.18(1.02, 1.37)	0.03	73.5	<0.01
rs2074196	5	11,019/11,672	1.31(1.23, 1.39)	<0.01	39.7	0.16	1.75(1.54, 1.99)	<0.01	26.7	0.24	1.33(1.22, 1.46)	<0.01	0.0	0.50	1.51(1.37, 1.66)	<0.01	6.4	0.37	1.37(1.27, 1.49)	<0.01	39.9	0.16

CI: confidence interval; T2DM: type 2 diabetes mellitus. ^a^Number of comparisons. ^b^*P* value of *Z*-test for the significant test. ^c^*P* value of *Q*-test for the between-study heterogeneity test.

## Supplementary Materials

Supplementary Materials Supplementary Fig. 1: forest plots of overall analysis between the *KCNQ1* polymorphisms and the risk of T2DM in homozygote comparison. Supplementary Fig. 2: funnel plots of overall analysis between the *KCNQ1* polymorphisms and the risk of T2DM in homozygote comparison. Supplementary Fig. 3: trial sequential analysis of the association between the *KCNQ1* polymorphisms and the risk of T2DM in allelic comparison. Supplementary Fig. 4: genotype-based mRNA expression alteration for *KCNQ1* rs2237892 polymorphism in subcutaneous adipose based on data from the GTEx Portal database (https://www.gtexportal.org/home/). Supplementary Fig. 5: genotype-based mRNA expression alteration for *KCNQ1* rs2283228 polymorphism in subcutaneous adipose based on data from the GTEx Portal database (https://www.gtexportal.org/home/). Supplementary Fig. 6: genotype-based mRNA expression alteration for *KCNQ1* rs231362 polymorphism in cultured fibroblasts based on data from the GTEx Portal database (https://www.gtexportal.org/home/). Supplementary Table 1: score of quality assessment. Supplementary Table 2: *KCNQ1* polymorphism genotype distribution among T2DM cases and controls of the included studies. Supplementary Table 3: stratified analysis of seven *KCNQ1* gene polymorphisms on T2DM. Supplementary Table 4: the metaregression analysis of homozygous *KCNQ1* gene on T2DM. Supplementary Table 5: publication bias tests (Egger's funnel plot for publication bias test) for seven *KCNQ1* gene polymorphisms.
