# Supplementary material for: Associations of KCNQ1 Polymorphisms with the Risk of Type 2 Diabetes Mellitus: An Updated Meta-Analysis with Trial Sequential Analysis
Source: J Diabetes Res. 2020 Jul 3;2020:7145139. doi: 10.1155/2020/7145139 (PMC7362295; doi:10.1155/2020/7145139)
Supplement: Supplementary Materials — Supplementary Fig. 1: forest plots of overall analysis between the KCNQ1 polymorphisms and the risk of T2DM in homozygote comparison. Supplementary Fig. 2: funnel plots of overall analysis between the KCNQ1 polymorphisms and the risk of T2DM in homozygote comparison. Supplementary Fig. 3: trial sequential analysis of the association between the KCNQ1 polymorphisms and the risk of T2DM in allelic comparison. Supplementary Fig. 4: genotype-based mRNA expression alteration for KCNQ1 rs2237892 polymorphism in subcutaneous adipose based on data from the GTEx Portal database (https://www.gtexportal.org/home/). Supplementary Fig. 5: genotype-based mRNA expression alteration for KCNQ1 rs2283228 polymorphism in subcutaneous adipose based on data from the GTEx Portal database (https://www.gtexportal.org/home/). Supplementary Fig. 6: genotype-based mRNA expression alteration for KCNQ1 rs231362 polymorphism in cultured fibroblasts based on data from the GTEx Portal database (https://www.gtexportal.org/home/). Supplementary Table 1: score of quality assessment. Supplementary Table 2: KCNQ1 polymorphism genotype distribution among T2DM cases and controls of the included studies. Supplementary Table 3: stratified analysis of seven KCNQ1 gene polymorphisms on T2DM. Supplementary Table 4: the metaregression analysis of homozygous KCNQ1 gene on T2DM. Supplementary Table 5: publication bias tests (Egger's funnel plot for publication bias test) for seven KCNQ1 gene polymorphisms. [file 7145139.f1.pdf]

Supplementary Material

supplementary figure legends

supplementary Fig. 1 Forest plots of overall analysis between *KCNQ1* polymorphisms and the risk of T2DM in homozygote comparison

supplementary Fig. 2 Funnel plots of overall analysis between *KCNQ1* polymorphisms and the risk of T2DM in homozygote comparison

supplementary Fig. 3 Trial sequential analysis of the association between *KCNQ1* polymorphisms and the risk of T2DM in allelic comparison

supplementary Fig. 4 Genotype - based mRNA expression alteration for KCNQ1 rs2237892 polymorphism in adipose - subcutaneous based on data from the GTEx portal database (https://www. gtexportal.org/home/)

supplementary Fig. 5 Genotype - based mRNA expression alteration for KCNQ1 rs2283228 polymorphism in adipose - subcutaneous based on data from the GTEx portal database (https://www. gtexportal.org/home/)

supplementary Fig. 6 Genotype - based mRNA expression alteration for KCNQ1 rs231362 polymorphism in cells - cultured fibroblasts based on data from the GTEx portal database (https://www. gtexportal.org/home/)

a

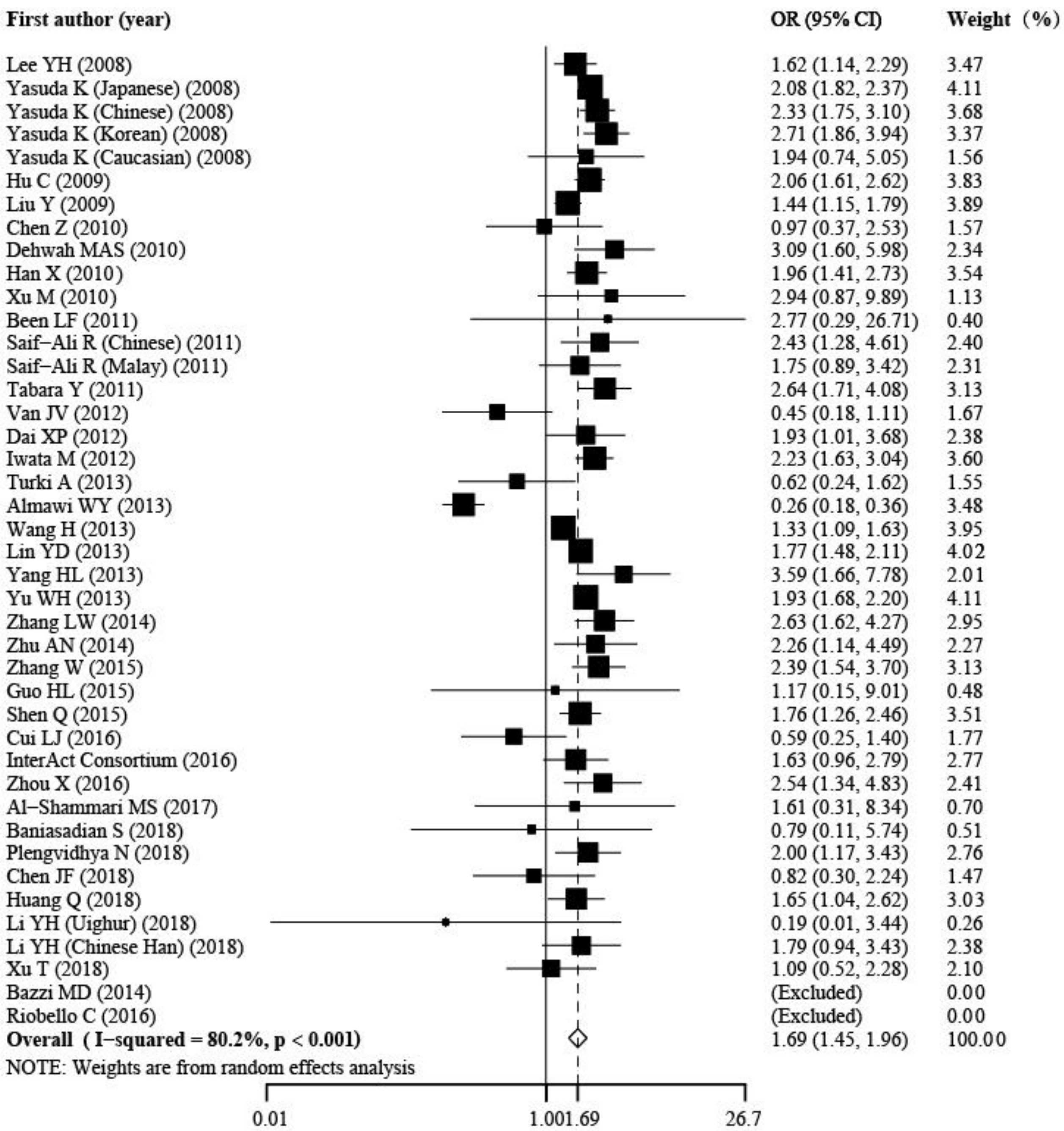
First author (year)
OR (95% CI)
Weight （%）
Lee YH (2008) 1.62 (1.14, 2.29) 3.47
Yasuda K (Japanese) (2008) 2.08 (1.82, 2.37) 4.11
Yasuda K (Chinese) (2008) 2.33 (1.75, 3.10) 3.68
Yasuda K (Korean) (2008) 2.71 (1.86, 3.94) 3.37
Yasuda K (Caucasian) (2008) 1.94 (0.74, 5.05) 1.56
Hu C (2009) 2.06 (1.61, 2.62) 3.83
Liu Y (2009) 1.44 (1.15, 1.79) 3.89
Chen Z (2010) 0.97 (0.37, 2.53) 1.57
Dehwah MAS (2010) 3.09 (1.60, 5.98) 2.34
Han X (2010) 1.96 (1.41, 2.73) 3.54
Xu M (2010) 2.94 (0.87, 9.89) 1.13
Been LF (2011) 2.77 (0.29, 26.71) 0.40
Saif−Ali R (Chinese) (2011) 2.43 (1.28, 4.61) 2.40
Saif−Ali R (Malay) (2011) 1.75 (0.89, 3.42) 2.31
Tabara Y (2011) 2.64 (1.71, 4.08) 3.13
Van JV (2012) 0.45 (0.18, 1.11) 1.67
Dai XP (2012) 1.93 (1.01, 3.68) 2.38
Iwata M (2012) 2.23 (1.63, 3.04) 3.60
Turki A (2013) 0.62 (0.24, 1.62) 1.55
Almawi WY (2013) 0.26 (0.18, 0.36) 3.48
Wang H (2013) 1.33 (1.09, 1.63) 3.95
Lin YD (2013) 1.77 (1.48, 2.11) 4.02
Yang HL (2013) 3.59 (1.66, 7.78) 2.01
Yu WH (2013) 1.93 (1.68, 2.20) 4.11
Zhang LW (2014) 2.63 (1.62, 4.27) 2.95
Zhu AN (2014) 2.26 (1.14, 4.49) 2.27
Zhang W (2015) 2.39 (1.54, 3.70) 3.13
Guo HL (2015) 1.17 (0.15, 9.01) 0.48
Shen Q (2015) 1.76 (1.26, 2.46) 3.51
Cui LJ (2016) 0.59 (0.25, 1.40) 1.77
InterAct Consortium (2016) 1.63 (0.96, 2.79) 2.77
Zhou X (2016) 2.54 (1.34, 4.83) 2.41
Al−Shammari MS (2017) 1.61 (0.31, 8.34) 0.70
Baniasadian S (2018) 0.79 (0.11, 5.74) 0.51
Plengvidhya N (2018) 2.00 (1.17, 3.43) 2.76
Chen JF (2018) 0.82 (0.30, 2.24) 1.47
Huang Q (2018) 1.65 (1.04, 2.62) 3.03
Li YH (Uighur) (2018) 0.19 (0.01, 3.44) 0.26
Li YH (Chinese Han) (2018) 1.79 (0.94, 3.43) 2.38
Xu T (2018) 1.09 (0.52, 2.28) 2.10
Bazzi MD (2014) (Excluded) 0.00
Riobello C (2016) (Excluded) 0.00
Overall (I−squared = 80.2%, p < 0.001) 1.69 (1.45, 1.96) 100.00
NOTE: Weights are from random effects analysis
0.01
1.001.69
26.7

b

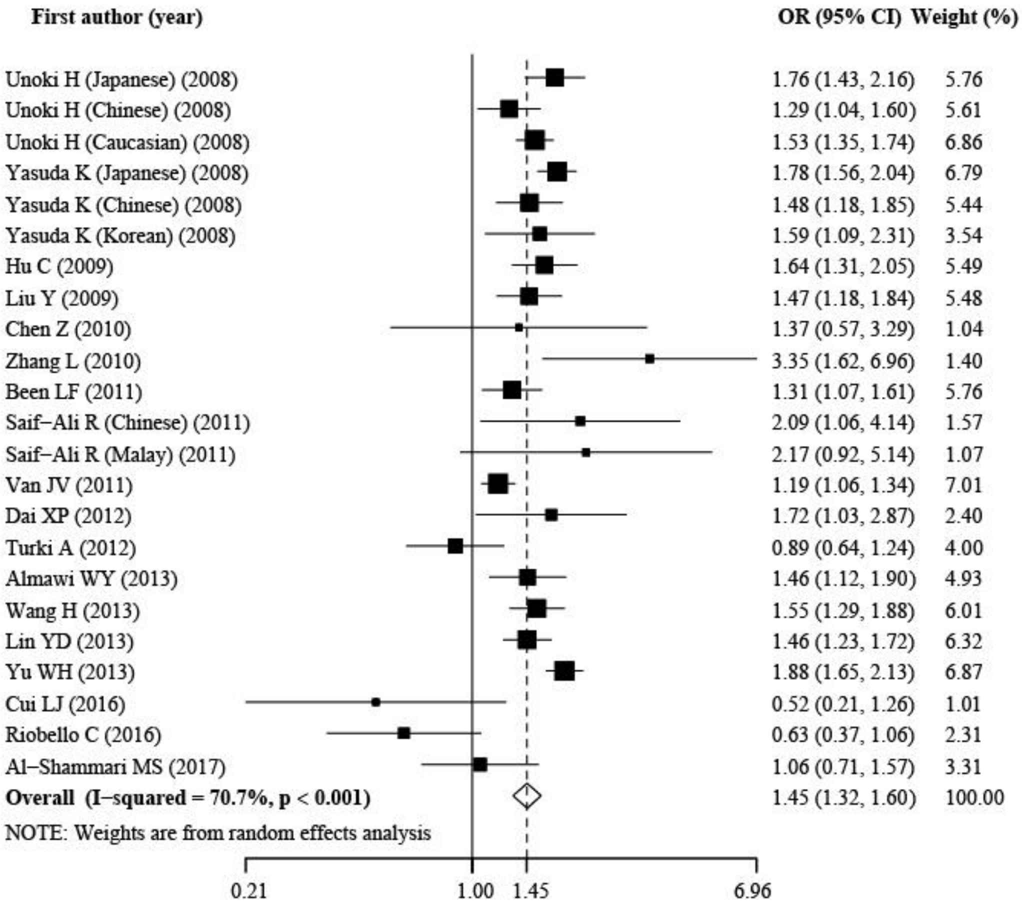
First author (year)
OR (95% CI) Weight (%)
Unoki H (Japanese) (2008) 1.76 (1.43, 2.16) 5.76
Unoki H (Chinese) (2008) 1.29 (1.04, 1.60) 5.61
Unoki H (Caucasian) (2008) 1.53 (1.35, 1.74) 6.86
Yasuda K (Japanese) (2008) 1.78 (1.56, 2.04) 6.79
Yasuda K (Chinese) (2008) 1.48 (1.18, 1.85) 5.44
Yasuda K (Korean) (2008) 1.59 (1.09, 2.31) 3.54
Hu C (2009) 1.64 (1.31, 2.05) 5.49
Liu Y (2009) 1.47 (1.18, 1.84) 5.48
Chen Z (2010) 1.37 (0.57, 3.29) 1.04
Zhang L (2010) 3.35 (1.62, 6.96) 1.40
Been LF (2011) 1.31 (1.07, 1.61) 5.76
Saif−Ali R (Chinese) (2011) 2.09 (1.06, 4.14) 1.57
Saif−Ali R (Malay) (2011) 2.17 (0.92, 5.14) 1.07
Van JV (2011) 1.19 (1.06, 1.34) 7.01
Dai XP (2012) 1.72 (1.03, 2.87) 2.40
Turki A (2012) 0.89 (0.64, 1.24) 4.00
Almawi WY (2013) 1.46 (1.12, 1.90) 4.93
Wang H (2013) 1.55 (1.29, 1.88) 6.01
Lin YD (2013) 1.46 (1.23, 1.72) 6.32
Yu WH (2013) 1.88 (1.65, 2.13) 6.87
Cui LJ (2016) 0.52 (0.21, 1.26) 1.01
Riobello C (2016) 0.63 (0.37, 1.06) 2.31
Al−Shammari MS (2017) 1.06 (0.71, 1.57) 3.31
Overall (I−squared = 70.7%, p < 0.001) 1.45 (1.32, 1.60) 100.00
NOTE: Weights are from random effects analysis
0.21
1.00
1.45
6.96

c

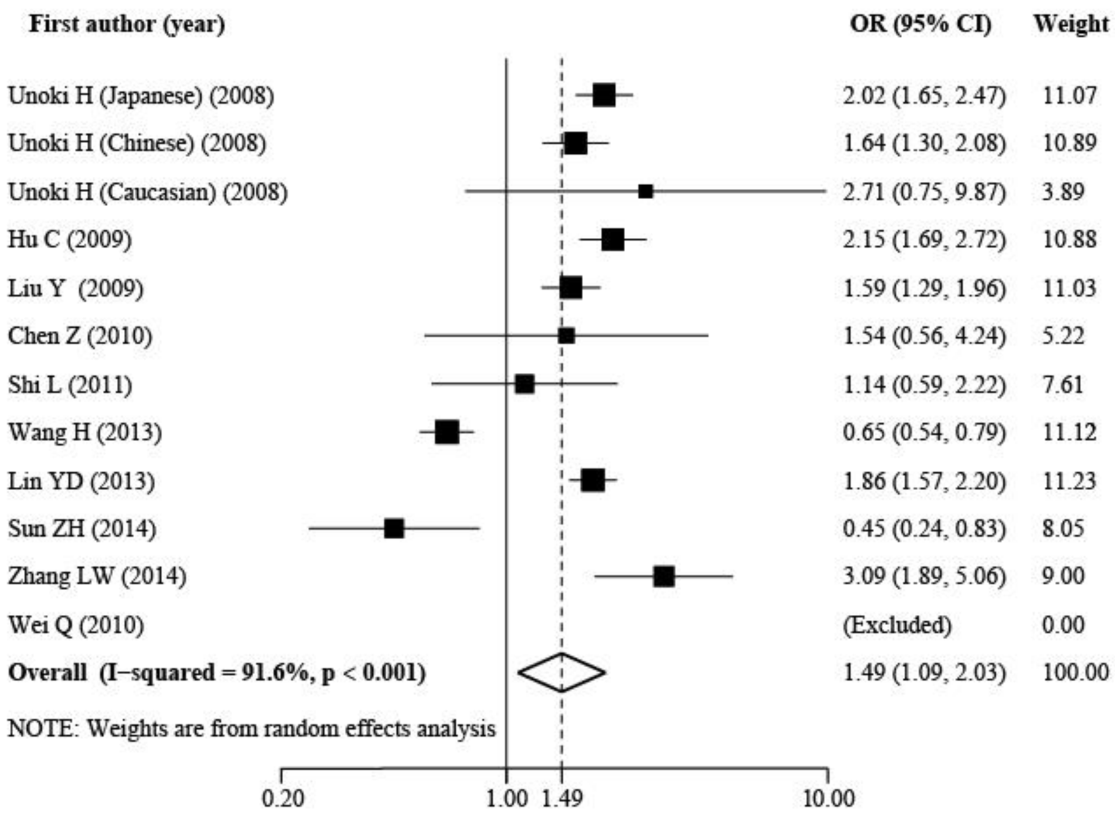
First author (year)
OR (95% CI)
Weight
Unoki H (Japanese) (2008)
2.02 (1.65, 2.47)
11.07
Unoki H (Chinese) (2008)
1.64 (1.30, 2.08)
10.89
Unoki H (Caucasian) (2008)
2.71 (0.75, 9.87)
3.89
Hu C (2009)
2.15 (1.69, 2.72)
10.88
Liu Y (2009)
1.59 (1.29, 1.96)
11.03
Chen Z (2010)
1.54 (0.56, 4.24)
5.22
Shi L (2011)
1.14 (0.59, 2.22)
7.61
Wang H (2013)
0.65 (0.54, 0.79)
11.12
Lin YD (2013)
1.86 (1.57, 2.20)
11.23
Sun ZH (2014)
0.45 (0.24, 0.83)
8.05
Zhang LW (2014)
3.09 (1.89, 5.06)
9.00
Wei Q (2010)
(Excluded)
0.00
Overall (I−squared = 91.6%, p < 0.001)
1.49 (1.09, 2.03)
100.00
NOTE: Weights are from random effects analysis
0.20
1.00
1.49
10.00

d

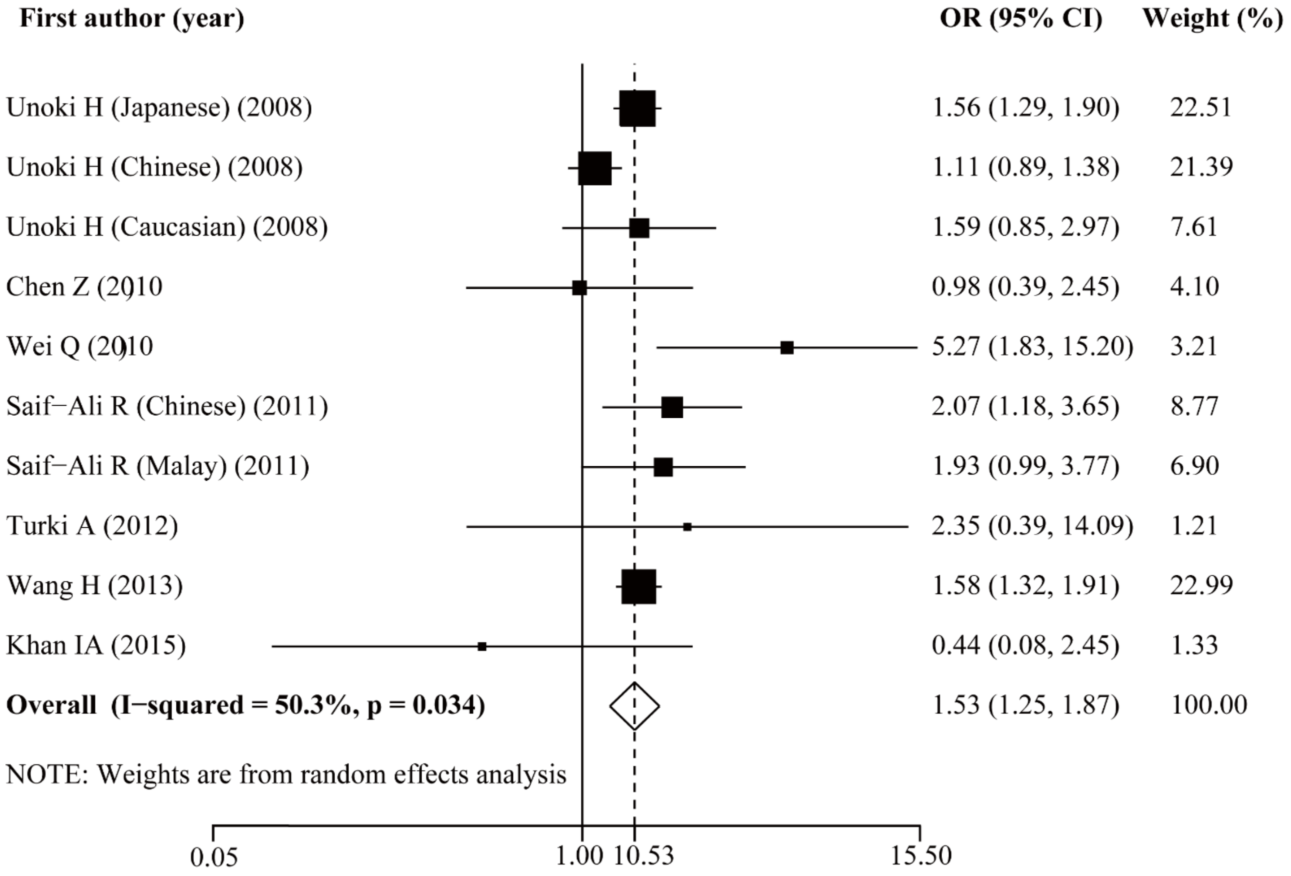

First author (year)
OR (95% CI)
Weight (%)
Unoki H (Japanese) (2008)
1.56 (1.29, 1.90)
22.51
Unoki H (Chinese) (2008)
1.11 (0.89, 1.38)
21.39
Unoki H (Caucasian) (2008)
1.59 (0.85, 2.97)
7.61
Chen Z (2010)
0.98 (0.39, 2.45)
4.10
Wei Q (2010)
5.27 (1.83, 15.20)
3.21
Saif−Ali R (Chinese) (2011)
2.07 (1.18, 3.65)
8.77
Saif−Ali R (Malay) (2011)
1.93 (0.99, 3.77)
6.90
Turki A (2012)
2.35 (0.39, 14.09)
1.21
Wang H (2013)
1.58 (1.32, 1.91)
22.99
Khan IA (2015)
0.44 (0.08, 2.45)
1.33
Overall (I−squared = 50.3%, p = 0.034)
1.53 (1.25, 1.87)
100.00
NOTE: Weights are from random effects analysis
0.05
1.00
10.53
15.50

e

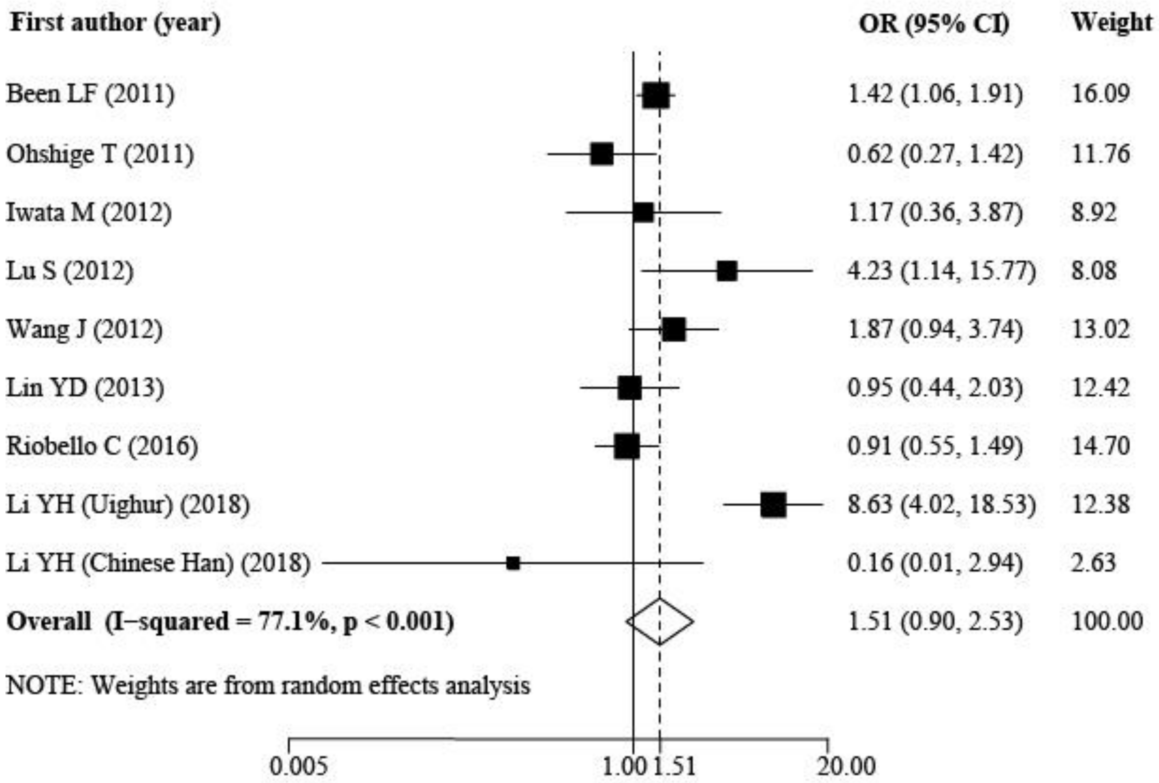
First author (year)
OR (95% CI)
Weight
Been LF (2011)
1.42 (1.06, 1.91)
16.09
Ohshige T (2011)
0.62 (0.27, 1.42)
11.76
Iwata M (2012)
1.17 (0.36, 3.87)
8.92
Lu S (2012)
4.23 (1.14, 15.77)
8.08
Wang J (2012)
1.87 (0.94, 3.74)
13.02
Lin YD (2013)
0.95 (0.44, 2.03)
12.42
Riobello C (2016)
0.91 (0.55, 1.49)
14.70
Li YH (Uighur) (2018)
8.63 (4.02, 18.53)
12.38
Li YH (Chinese Han) (2018)
0.16 (0.01, 2.94)
2.63
Overall (I−squared = 77.1%, p < 0.001)
1.51 (0.90, 2.53)
100.00
NOTE: Weights are from random effects analysis
0.005
1.00
1.51
20.00

f

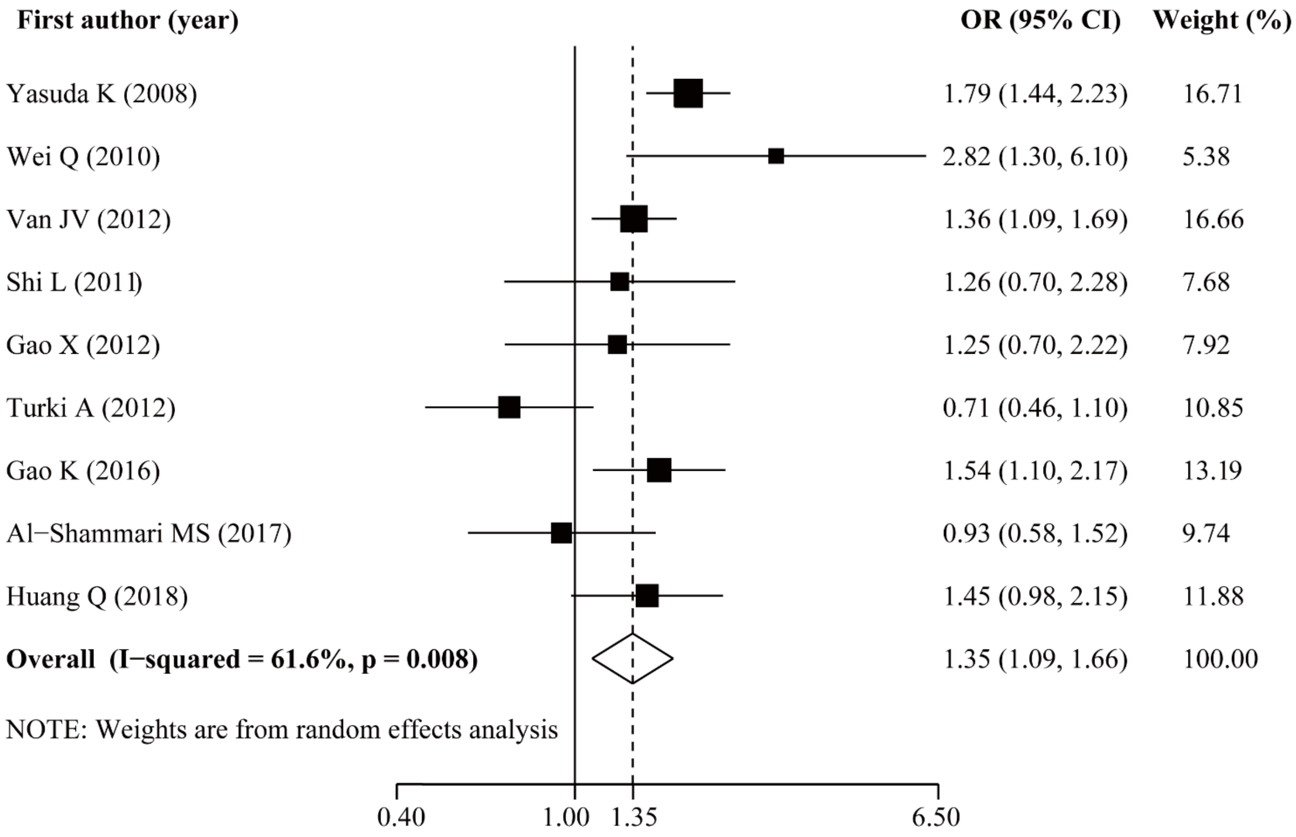
First author (year)
OR (95% CI)
Weight (%)
Yasuda K (2008)
1.79 (1.44, 2.23)
16.71
Wei Q (2010)
2.82 (1.30, 6.10)
5.38
Van JV (2012)
1.36 (1.09, 1.69)
16.66
Shi L (2011)
1.26 (0.70, 2.28)
7.68
Gao X (2012)
1.25 (0.70, 2.22)
7.92
Turki A (2012)
0.71 (0.46, 1.10)
10.85
Gao K (2016)
1.54 (1.10, 2.17)
13.19
Al−Shammari MS (2017)
0.93 (0.58, 1.52)
9.74
Huang Q (2018)
1.45 (0.98, 2.15)
11.88
Overall (I−squared = 61.6%, p = 0.008)
1.35 (1.09, 1.66)
100.00
NOTE: Weights are from random effects analysis
0.40
1.00
1.35
6.50

g

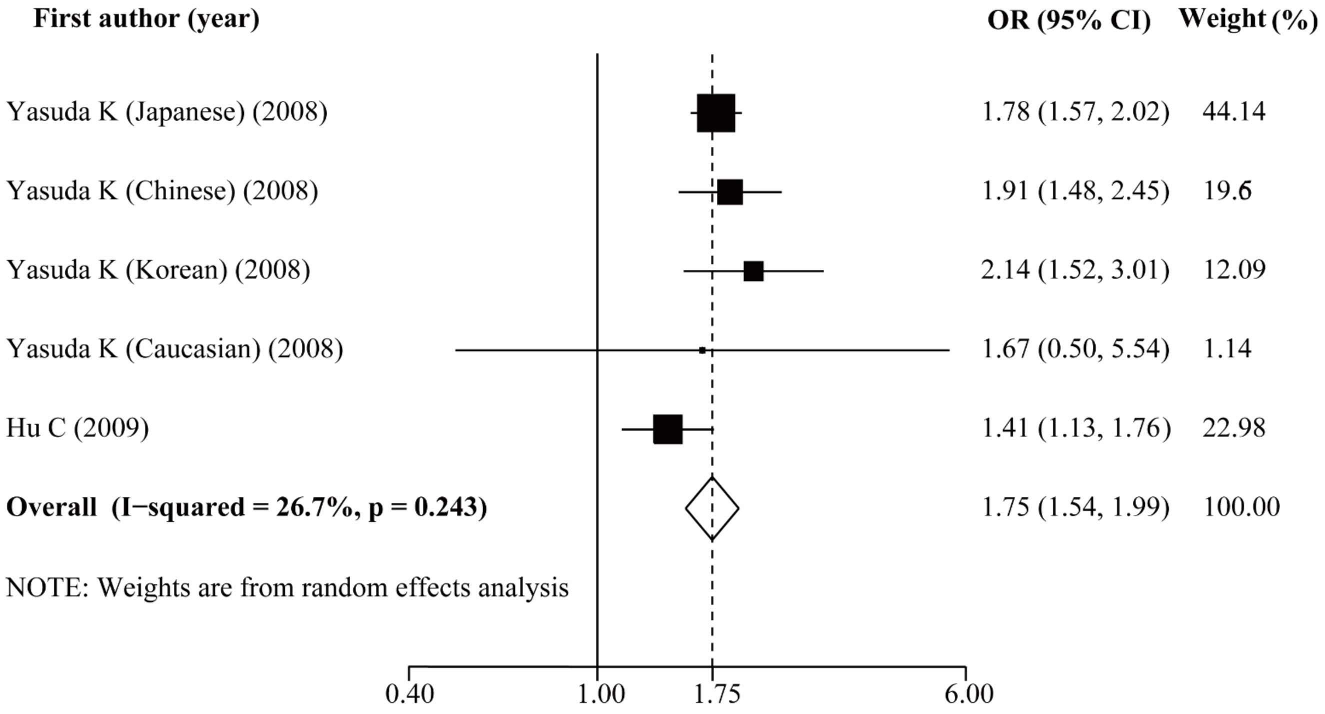

supplementary Fig. 1 Forest plots of overall analysis between *KCNQ1* polymorphisms and the risk of T2DM in homozygote comparison
a rs2237892, b rs2237895, c rs2237897, d rs2283228, e rs231362, f rs151290, g rs2074196

a

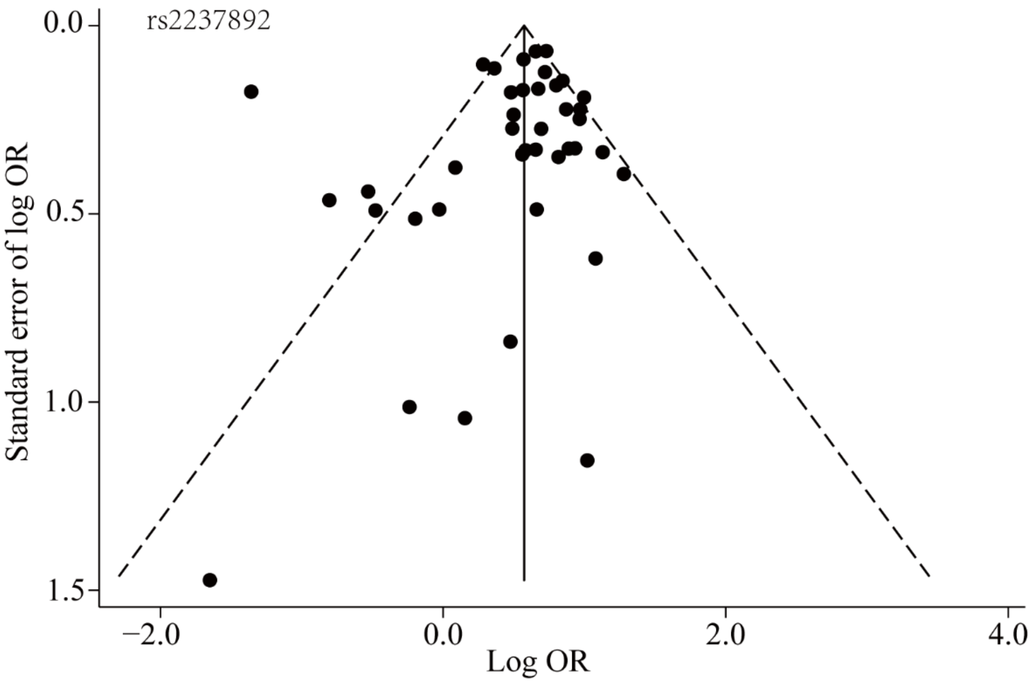
rs2237892
0.0
0.5
1.0
1.5
Standard error of log OR
−2.0
0.0
2.0
4.0
Log OR

b

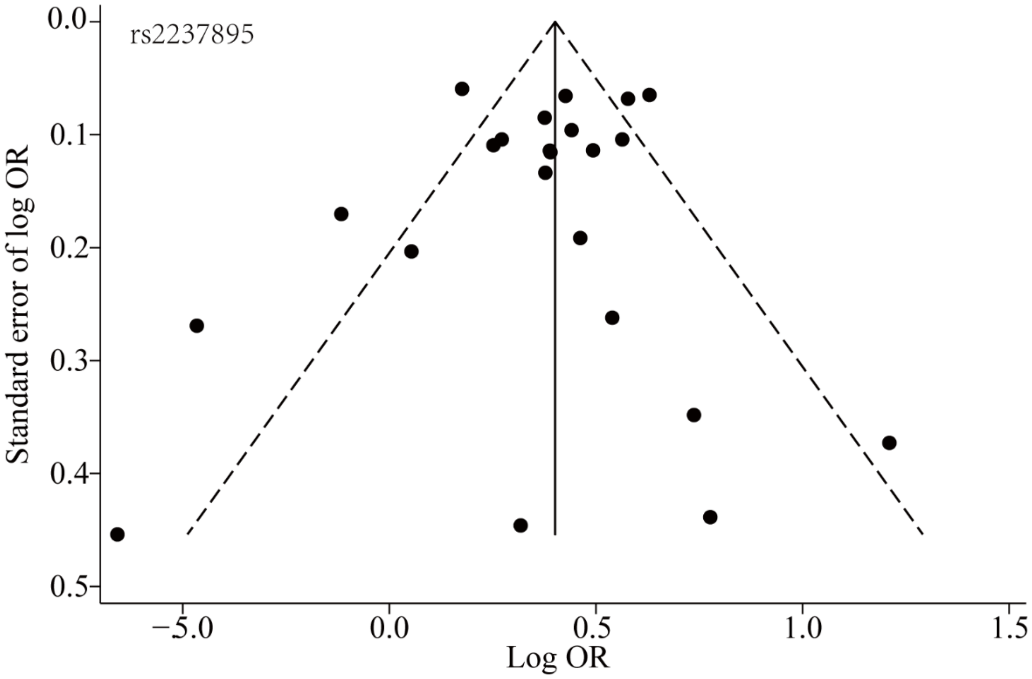
rs2237895
0.0
0.1
0.2
0.3
0.4
0.5
Standard error of log OR
−5.0
0.0
0.5
1.0
1.5
Log OR

c

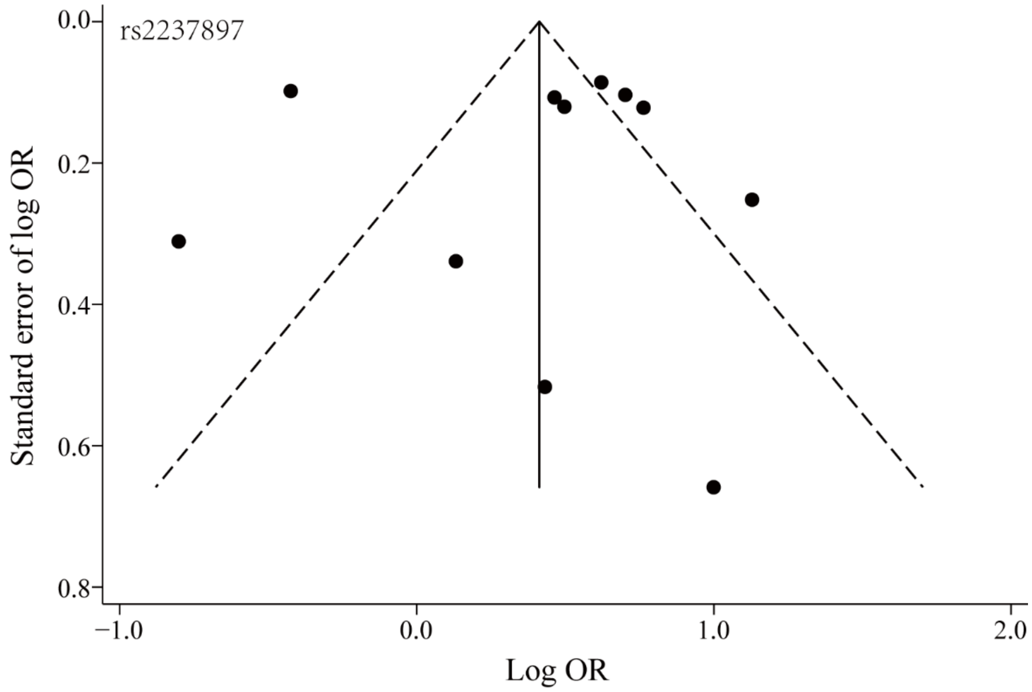
rs2237897
Standard error of log OR
0.0
0.2
0.4
0.6
0.8
−1.0
0.0
1.0
2.0
Log OR

d

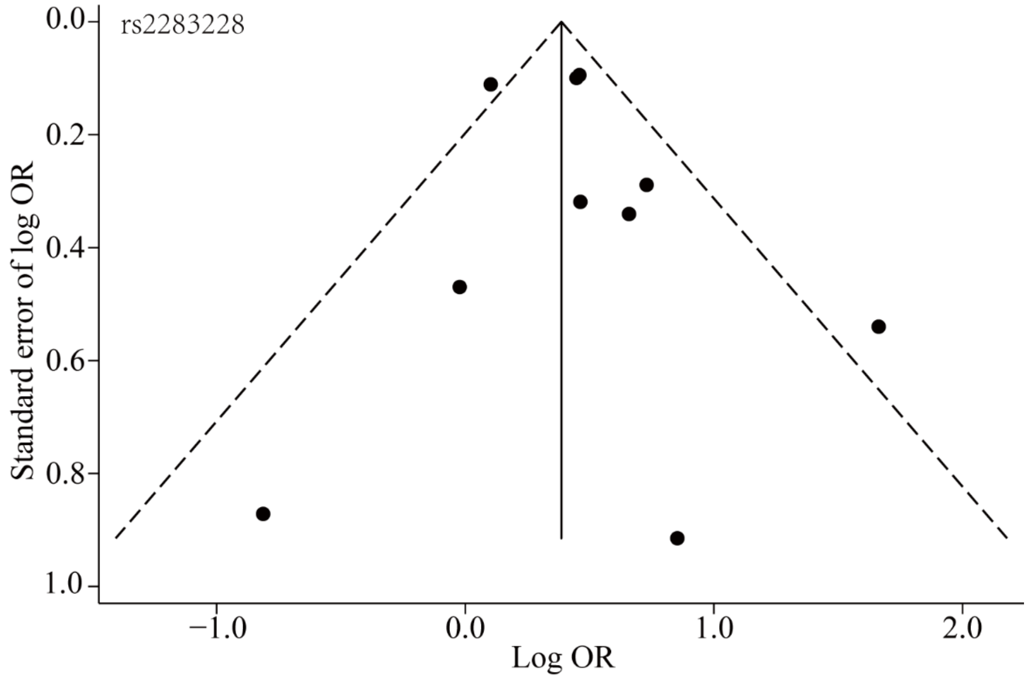
rs2283228
Standard error of log OR
0.0
0.2
0.4
0.6
0.8
1.0
−1.0
0.0
1.0
2.0
Log OR

e

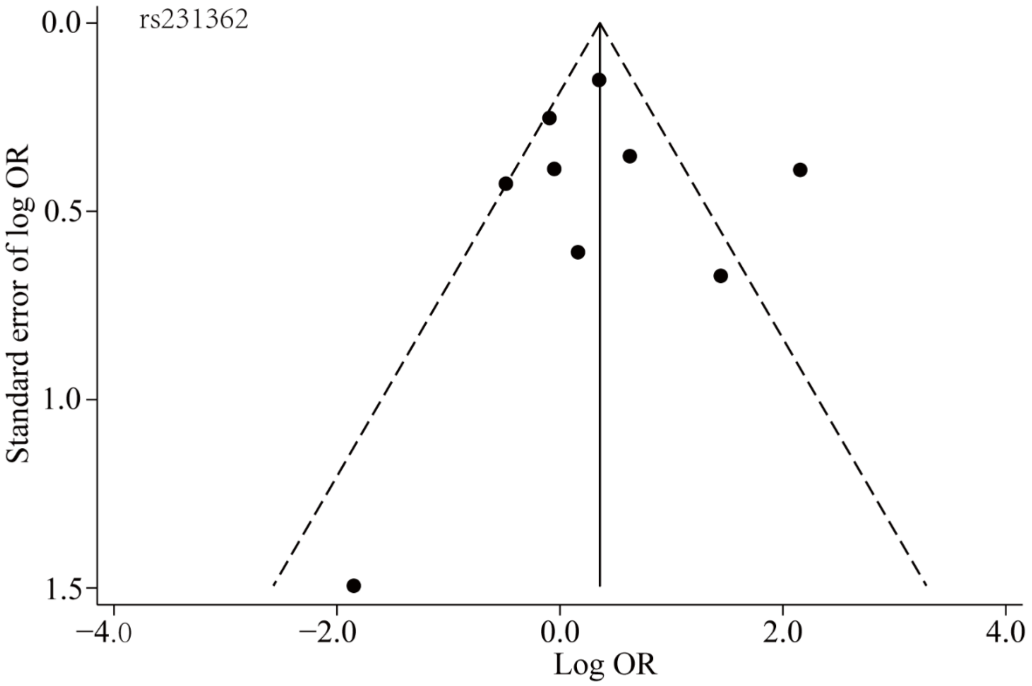

rs231362
0.0
0.5
1.0
1.5
Standard error of log OR
−4.0
−2.0
0.0
2.0
4.0
Log OR

f

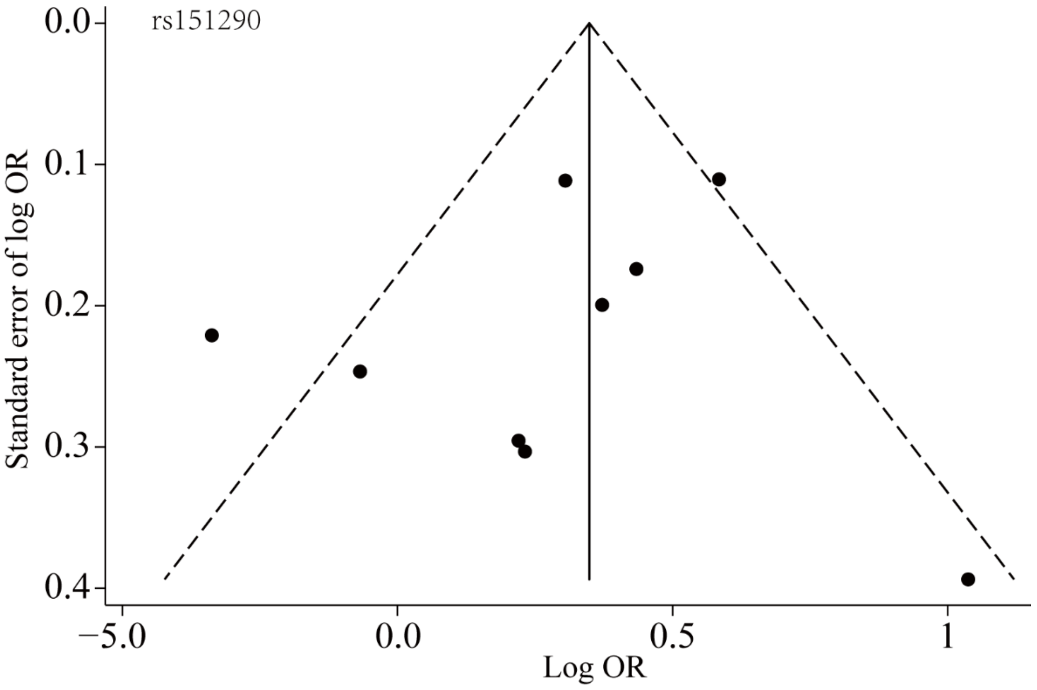

rs151290
0.0
0.1
0.2
0.3
0.4
Standard error of log OR
−5.0
0.0
0.5
1
Log OR

g

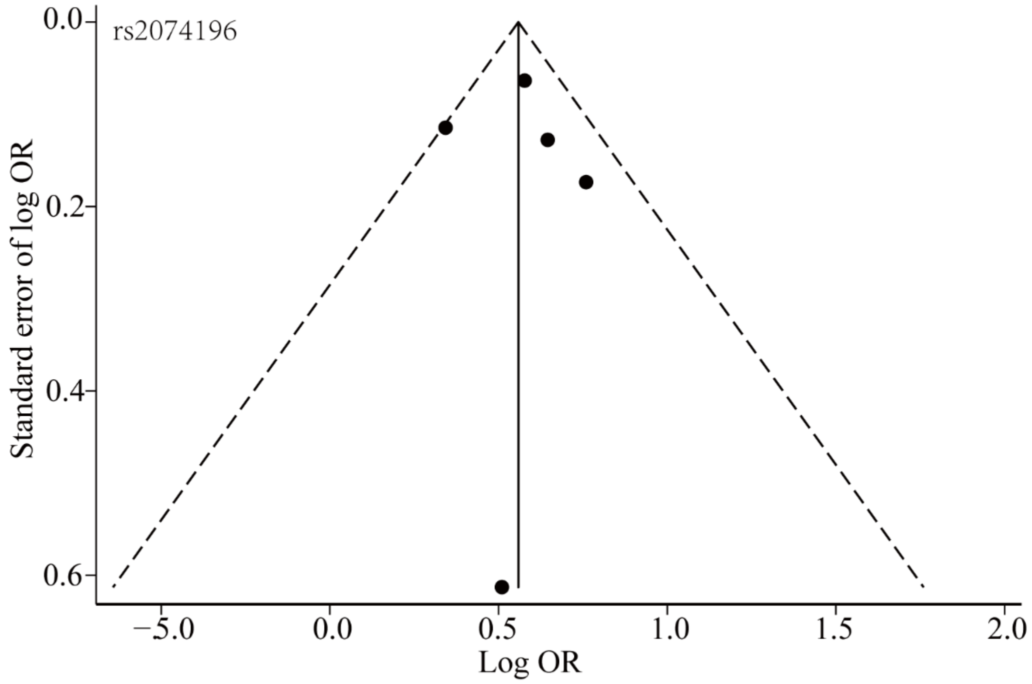

supplementary Fig. 2 Funnel plots of overall analysis between *KCNQ1* polymorphisms and the risk of T2DM in homozygote comparison
a rs2237892, b rs2237895, c rs2237897, d rs2283228, e rs231362, f rs151290, g rs2074196

a

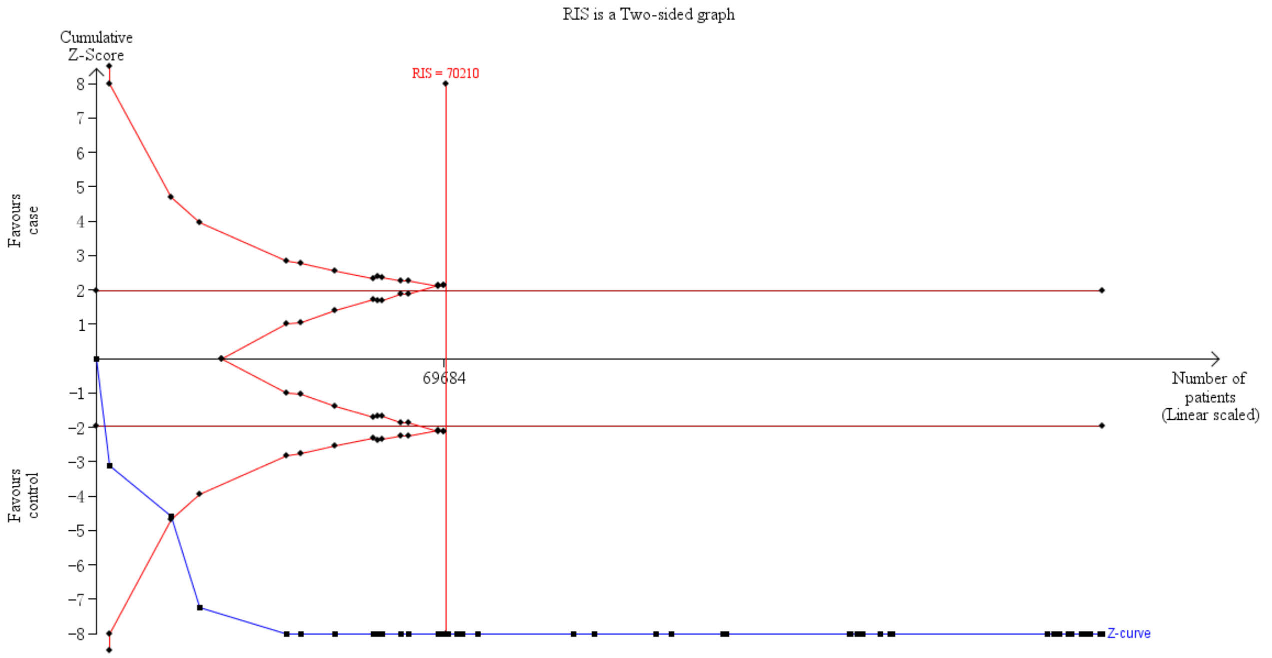
RIS is a Two-sided graph
Cumulative Z-Score
Favours case
Favours control
RIS = 70210
69684
Number of patients (Linear scaled)
Z-curve
8
7
6
5
4
3
2
1
-1
-2
-3
-4
-5
-6
-7
-8

b

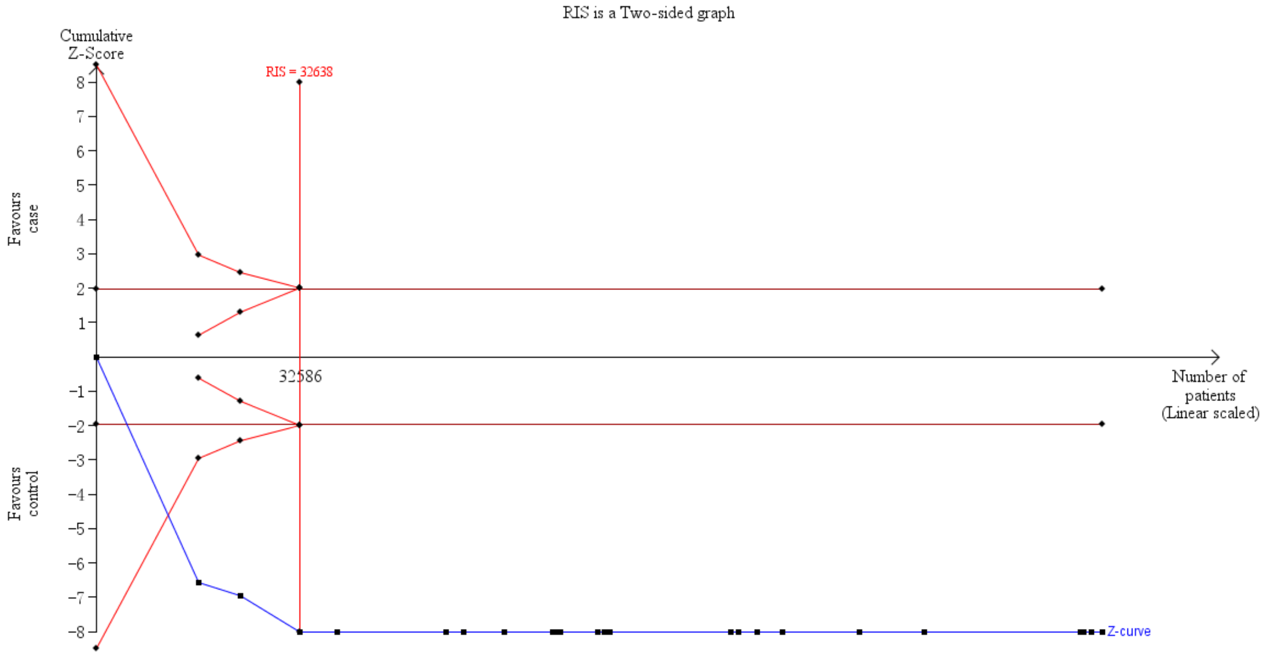
RIS is a Two-sided graph
Cumulative Z-Score
Favours case
Favours control
RIS = 32638
32586
Number of patients (Linear scaled)
Z-curve
8
7
6
5
4
3
2
1
-1
-2
-3
-4
-5
-6
-7
-8

c

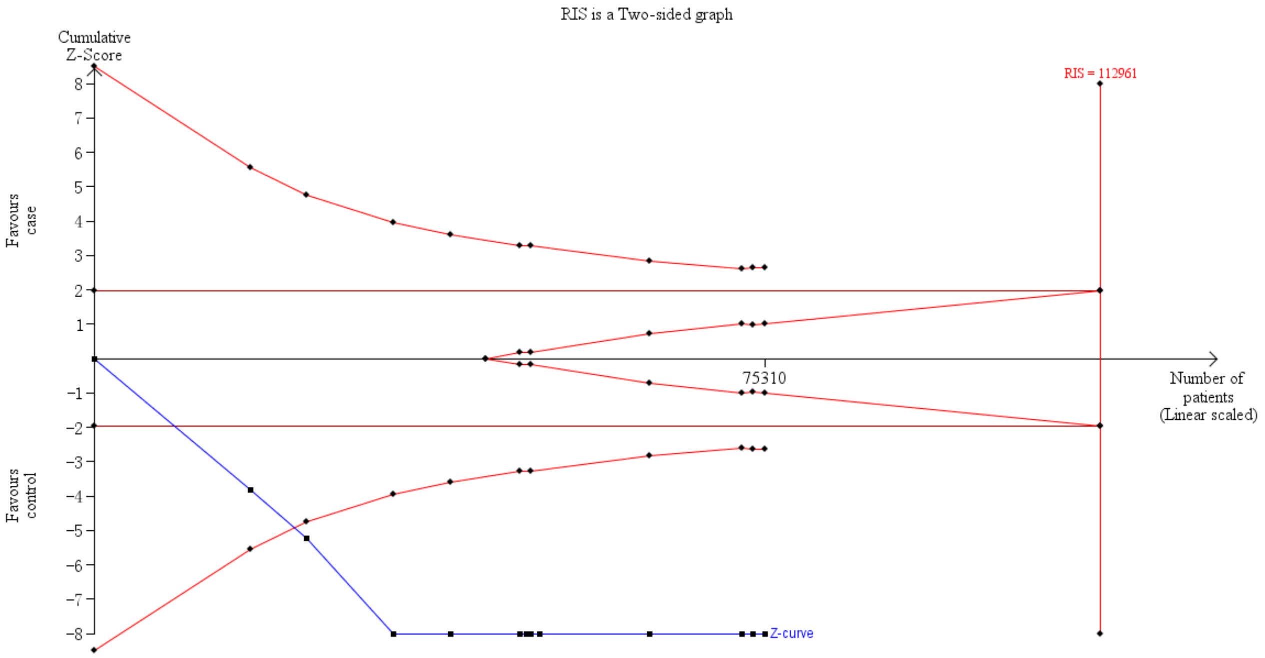

RIS is a Two-sided graph
Cumulative Z-Score
RIS = 112961
Favours case
Favours control
75310
Number of patients (Linear scaled)
Z-curve

d

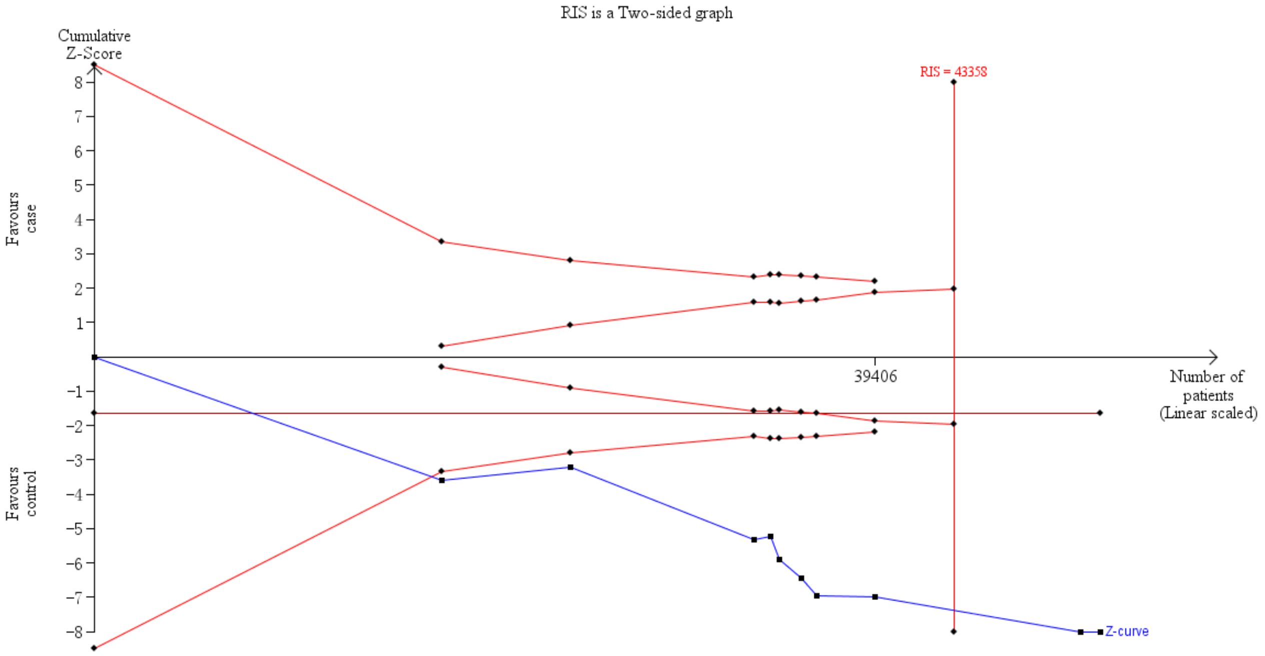

RIS is a Two-sided graph
Cumulative Z-Score
RIS = 43358
Favours case
Favours control
39406
Number of patients (Linear scaled)
Z-curve

e

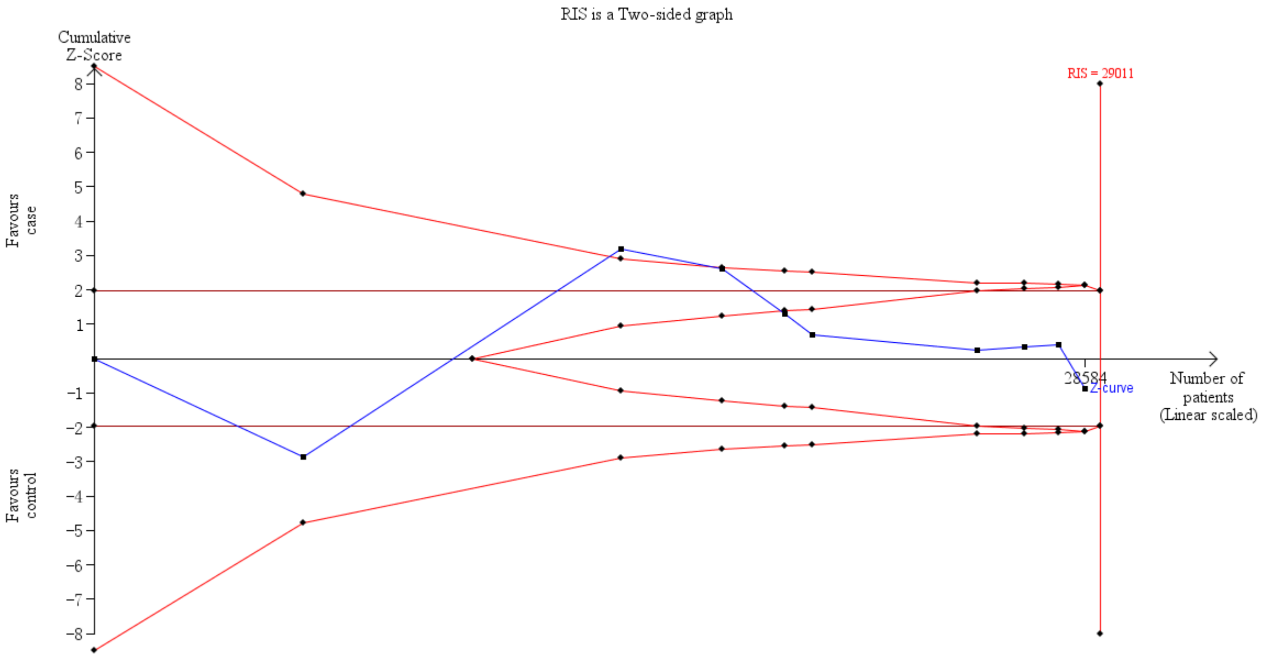
RIS is a Two-sided graph
Cumulative Z-Score
Favours case
Favours control
RIS = 29011
28584
Z-curve
Number of patients (Linear scaled)
8
7
6
5
4
3
2
1
-1
-2
-3
-4
-5
-6
-7
-8

f

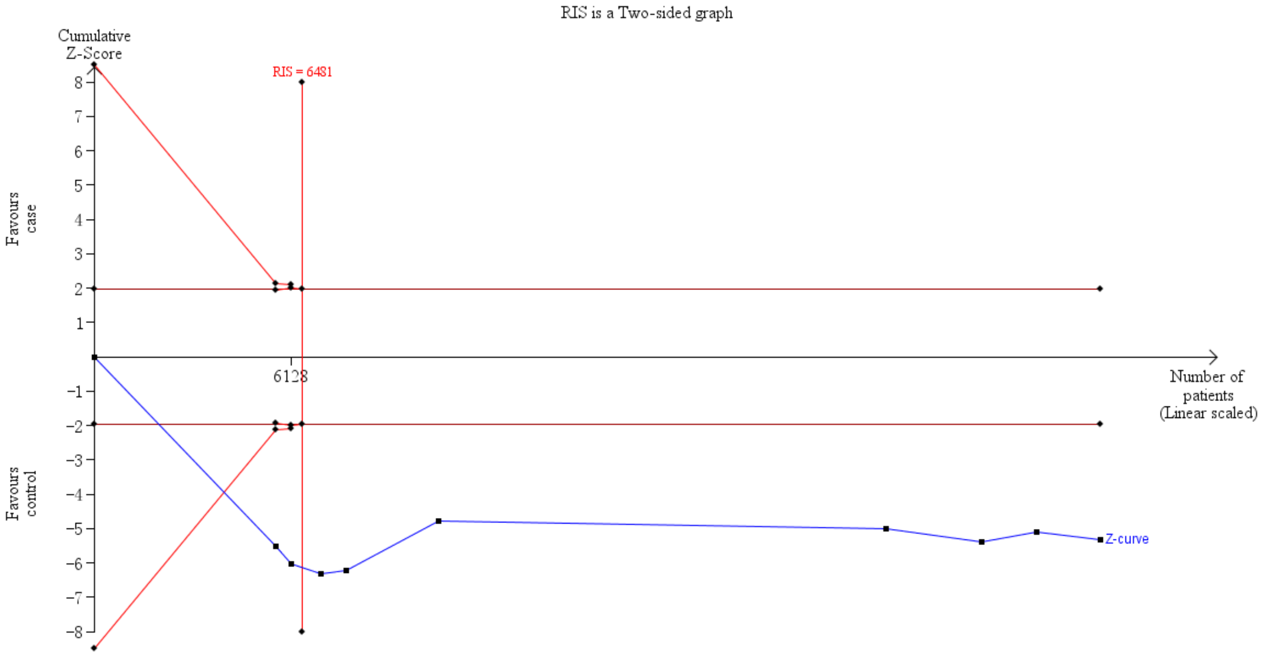
RIS is a Two-sided graph
Cumulative Z-Score
Favours case
Favours control
RIS = 6481
6128
Z-curve
Number of patients (Linear scaled)
8
7
6
5
4
3
2
1
-1
-2
-3
-4
-5
-6
-7
-8

g

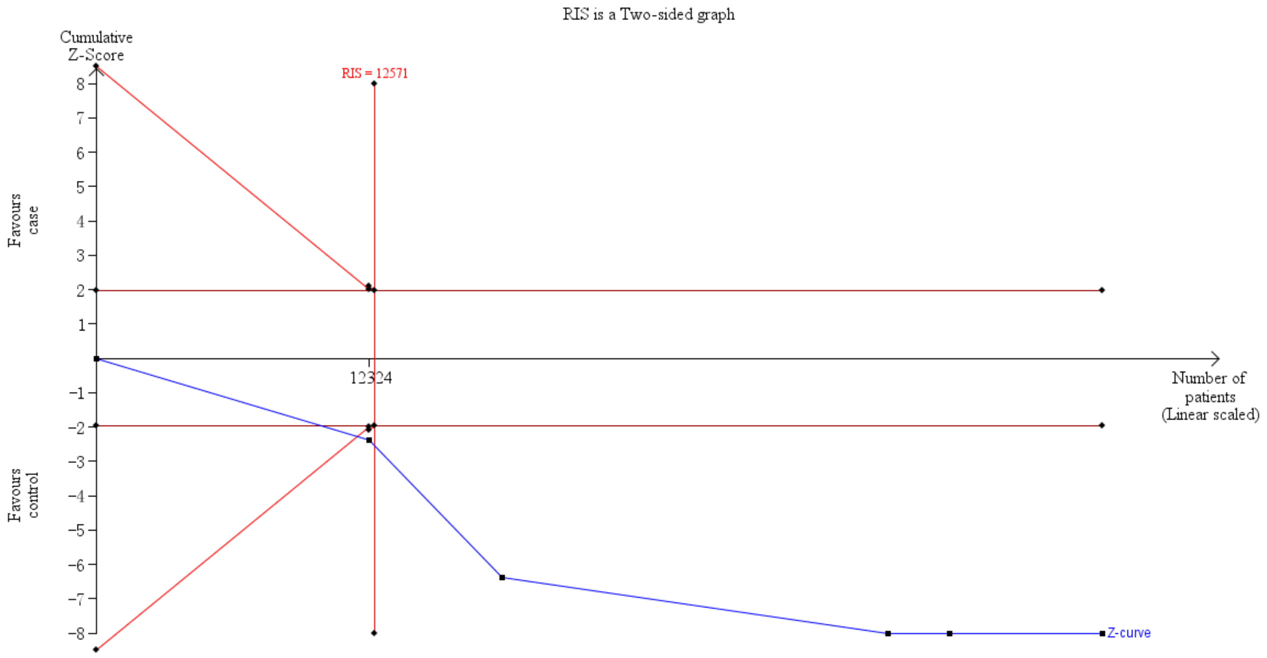

supplementary Fig. 3 Trial sequential analysis of the association between *KCNQ1* polymorphisms and the risk of T2DM in allelic comparison
a rs2237892, b rs2237895, c rs2237897, d rs2283228, e rs231362, f rs151290, g rs2074196

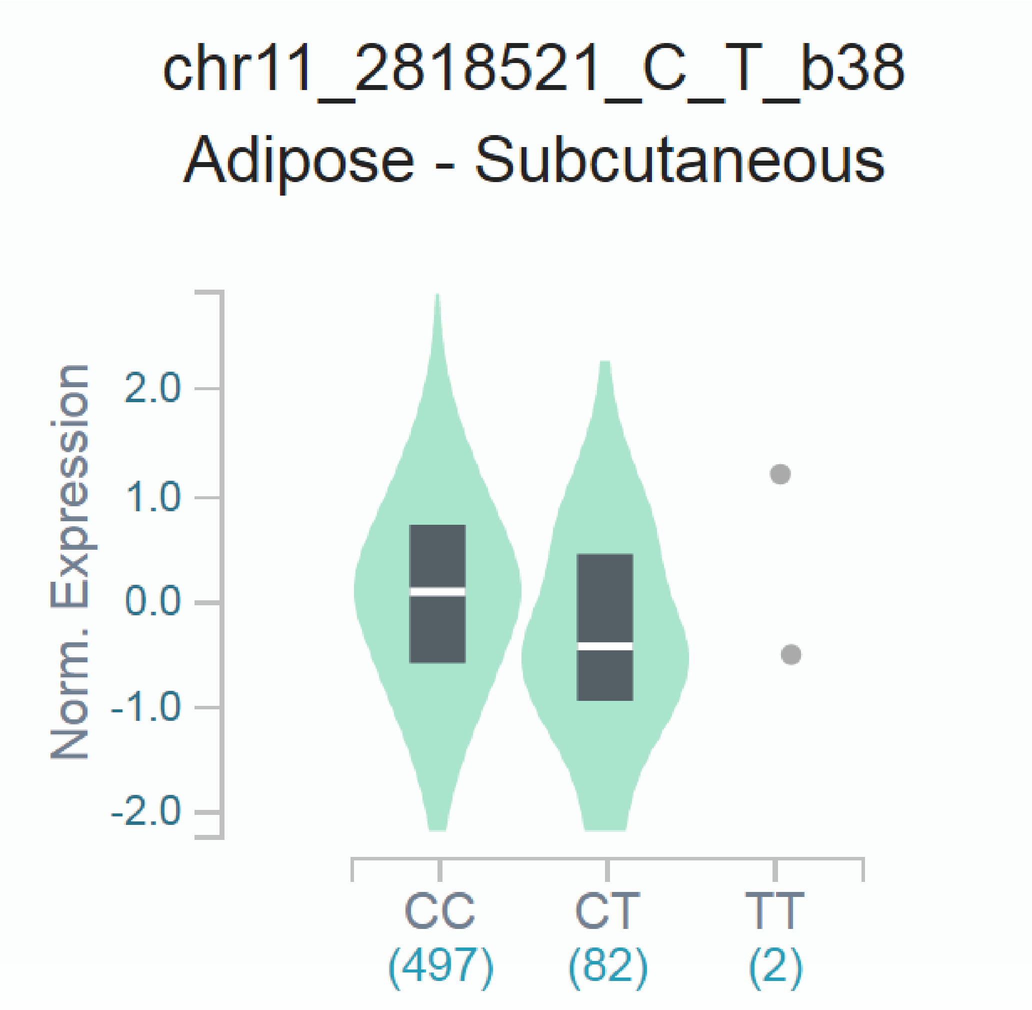

supplementary Fig. 4 Genotype - based mRNA expression alteration for KCNQ1 rs2237892 polymorphism in adipose - subcutaneous based on data from the GTEx portal database (https://www. gtexportal.org/home/)

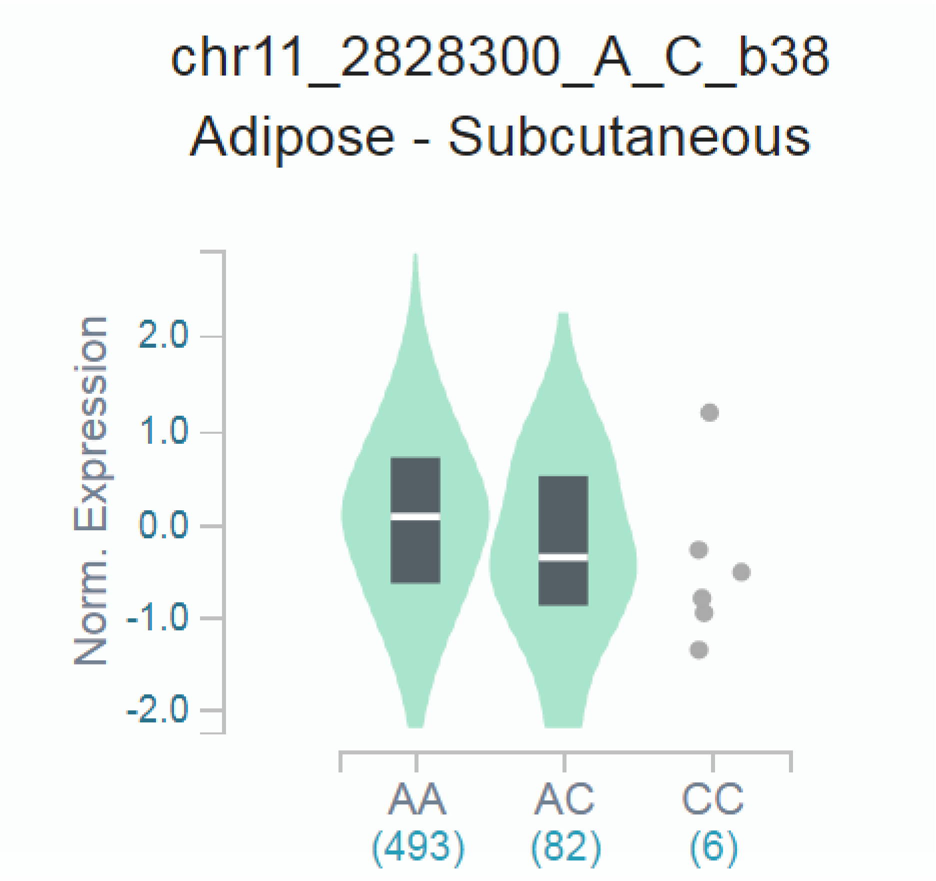

supplementary Fig. 5 Genotype - based mRNA expression alteration for KCNQ1 rs2283228 polymorphism in adipose - subcutaneous based on data from the GTEx portal database (https://www. gtexportal.org/home/)

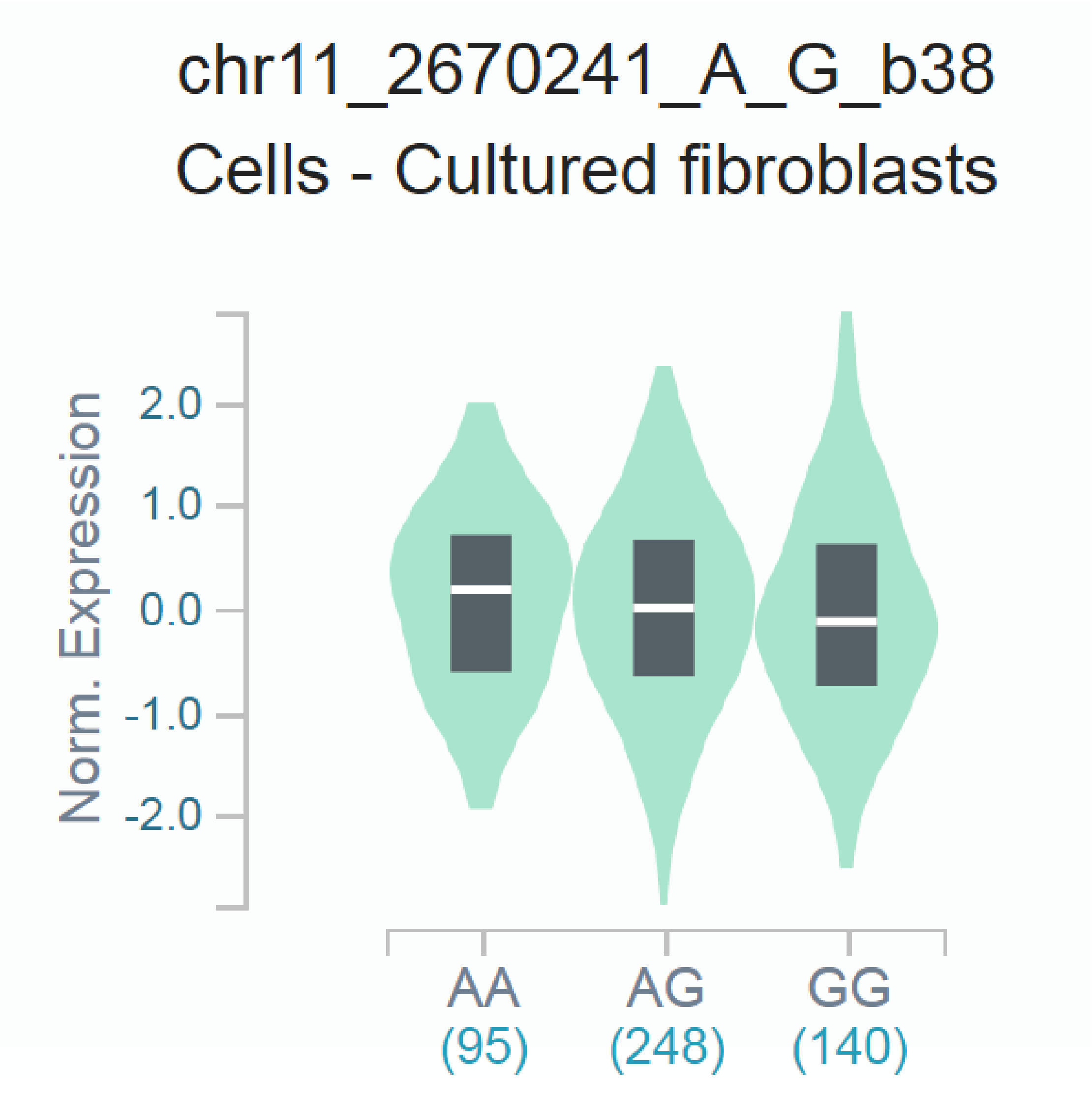

supplementary Fig. 6 Genotype - based mRNA expression alteration for KCNQ1 rs231362 polymorphism in cells - cultured fibroblasts based on data from the GTEx portal database (https://www. gtexportal.org/home/)

Supplementary Material

supplementary table legends

supplemental Table 1 Score of quality assessment

supplementary Table 2 *KCNQ1* polymorphisms genotype distribution among T2DM cases and controls of the included studies

supplemental Table 3 Stratified analysis of *KCNQ1* gene seven polymorphisms on T2DM

supplemental Table 4 The meta-regression analysis of *KCNQ1* gene homozygous on T2DM

supplementary Table 5 Publication bias tests (Egger's funnel plot for publication bias test) for *KCNQ1* gene seven polymorphisms

supplemental Table 1 Score of quality assessment

**Criteria**	**Score**
Representativeness of case	
Selected from population	2
Selected from hospital	1
No method of selection described	0
Representativeness of control	
Population-based	3
Blood donors	2
Hospital-based	1
Not described	0
Ascertainment of T2D case	
Glucose confirmation	2
By patient medical record	1
Not described	0
Control selection	
Controls matched with cases by age and sex	2
Controls matched with cases only by age or by sex	1
Not matched or not descried	0
Genotyping examination	
Genotyping done blindly and quality control	2
Only genotyping done blindly or quality control	1
Unblinded and without quality control	0
HWE	
HWE controls	1
HWE-deviated controls	0
Total sample size	
> 1000	3
501 - 1000	2
201 - 500	1
≤ 200	0

supplementary Table 2 *KCNQ1* polymorphisms genotype distribution among T2DM cases and controls of the included studies

First author	Year	Cases				Controls				P (HWE)
		WW	WM	MM	MAF (%)1	WW	WM	MM	MAF (%)[a]	
**rs2237892**										
Lee YH	2008	389	377	99	33.2	182	239	75	39.2	0.811
Yasuda K (Japanese)	2008	1980	1878	457	32.4	1582	2025	758	40.6	**0.012**
Yasuda K (Chinese)	2008	673	604	79	28.1	633	734	173	35.1	0.067
Yasuda K (Korean)	2008	334	366	52	31.3	237	291	100	39.1	0.500
Yasuda K (Caucasian)	2008	2466	287	6	5.4	2968	444	14	6.9	0.548
Hu C	2009	947	643	129	26.2	707	816	198	35.2	0.104
Liu Y	2009	902	813	165	30.4	853	919	224	34.2	0.318
Chen Z	2010	27	24	6	31.6	162	144	35	31.4	0.720
Dehwah MAS	2010	115	92	16	27.8	79	88	34	38.8	0.268
Han X	2010	525	396	69	27.0	415	437	107	33.9	0.616
Xu M	2010	30	33	3	29.5	272	300	80	35.3	0.846
Been LF	2011	1392	36	1	1.3	1505	68	3	2.3	**0.019**
Saif-Ali R (Chinese)	2011	183	99	18	22.5	113	90	27	31.3	0.171
Saif-Ali R (Malay)	2011	135	79	20	25.4	81	75	21	33.1	0.572
Tabara Y	2011	243	206	44	29.8	136	193	65	41.0	0.803
Van JV	2011	4149	348	14	4.2	4638	507	7	5.1	0.073
Dai XP	2012	233	112	22	21.3	110	82	20	28.8	0.412
Iwata M	2012	342	300	82	32.0	283	329	151	41.3	**0.002**
Turki A	2013	763	106	14	7.6	528	57	6	5.8	**0.003**
Almawi WY	2013	500	371	124	31.1	800	225	51	15.2	**<0.001**
Wang H	2013	1332	998	203	27.7	1210	1188	245	31.7	0.055
Lin YD	2013	1491	1174	234	28.3	1433	1431	397	34.1	0.174
Yang HL	2013	123	87	12	25.0	60	59	21	36.1	0.308
Yu WH	2013	4401	3424	620	27.6	1626	1803	441	34.7	0.080
Bazzi MD	2014	71	7	0	4.5	89	7	0	3.6	0.711
Zhang LW	2014	182	131	36	29.1	104	142	54	41.7	0.649
Zhu AN	2014	104	116	14	30.8	105	94	32	34.2	0.145
Zhang W	2015	274	217	39	27.8	194	192	66	35.8	0.104
Guo HL	2015	7	21	2	41.7	9	18	3	40.0	0.171
Shen Q	2015	441	374	66	28.7	395	382	104	33.5	0.430
Cui LJ	2016	39	46	15	38.0	53	35	12	29.5	0.113
InterAct Consortium	2016	6148	701	20	5.4	7721	946	41	5.9	**0.040**
Riobello C	2016	155	25	0	6.9	450	51	0	5.1	0.230
Zhou X	2016	148	136	21	29.2	72	102	26	38.5	0.276
Al-Shammari MS	2017	319	9	2	2.0	496	15	5	2.4	**<0.001**
Baniasadian S	2018	67	8	2	7.8	85	3	2	3.9	**<0.001**
Plengvidhya N	2018	285	192	23	23.8	254	205	41	28.7	0.968
Chen JF	2018	34	42	8	34.5	57	36	11	27.9	0.155
Huang Q	2018	250	220	36	28.9	215	231	51	33.5	0.336
Li YH (Uighur)	2018	210	68	6	14.1	84	15	0	7.6	0.415
Li YH (Chinese Han)	2018	144	128	21	29.0	88	97	23	34.4	0.628
Xu T	2018	31	45	24	46.5	32	41	27	47.5	0.075
**rs2237895**										
Unoki H (Japanese)	2008	1,225	1,633	577	40.6	567	580	152	34.0	0.843
Unoki H (Chinese)	2008	574	709	214	38.0	801	848	232	34.9	0.744
Unoki H (Caucasian)	2008	1,082	1,854	750	45.5	1,578	2,203	714	40.4	0.233
Yasuda K (Japanese)	2008	1546	2087	692	40.1	1939	1926	487	33.3	0.791
Yasuda K (Chinese)	2008	506	698	212	39.6	684	697	194	34.4	0.426
Yasuda K (Korean)	2008	305	359	86	35.4	304	270	54	30.1	0.585
Hu C	2009	636	759	234	37.7	770	699	173	31.8	0.444
Liu Y	2009	790	886	209	34.6	942	883	169	30.6	0.059
Chen Z	2010	23	26	8	36.8	162	138	41	32.3	0.172

Zhang L	2010	25	36	39	57.0	43	34	20	38.1	**0.011**
Been LF	2011	450	677	297	44.6	565	722	284	41.1	**0.045**
Saif-Ali R (Chinese)	2011	123	147	30	34.5	120	96	14	27.0	0.364
Saif-Ali R (Malay)	2011	123	91	20	28.0	107	62	8	22.0	0.795
Van JV	2011	1522	2158	869	42.8	1803	2516	863	40.9	0.768
Dai XP	2012	134	168	65	40.6	99	87	28	33.4	0.207
Turki A	2013	350	429	107	36.3	233	261	80	36.7	0.614
Almawi WY	2013	324	497	174	42.5	413	511	152	37.9	0.763
Wang H	2013	1038	1190	305	35.5	1270	1133	240	30.5	0.576
Lin YD	2013	1233	1289	374	35.2	1546	1390	322	31.2	0.711
Yu WH	2013	3242	4026	1285	38.6	1837	1705	388	31.6	0.793
Cui LJ	2016	40	49	11	35.5	32	51	17	42.5	0.664
Riobello C	2016	65	90	25	38.9	155	251	95	44.0	0.711
Al-Shammari MS	2017	122	150	58	40.3	202	223	91	39.2	**0.033**
**rs2237897**										
Unoki H (Japanese)	2008	1,615	1,574	333	31.8	476	646	198	39.5	0.379
Unoki H (Chinese)	2008	680	621	132	30.9	749	747	239	35.3	**0.017**
Unoki H (Caucasian)	2008	3,643	245	3	3.2	4,473	405	10	4.3	0.793
Hu C	2009	832	654	131	28.3	660	740	223	36.5	0.498
Liu Y	2009	873	822	183	31.6	813	912	271	36.4	0.549
Chen Z	2010	25	27	5	32.5	156	137	48	34.2	**0.048**
Wei Q	2010	70	63	0	23.7	26	80	0	37.7	**<0.001**
Shi L	2011	93	59	19	28.4	103	161	24	36.3	**<0.001**
Wang H	2013	211	1054	1268	70.9	302	1154	1187	66.7	0.397
Lin YD	2013	1440	1196	267	29.8	1308	1503	451	36.9	0.562
Sun ZH	2014	156	131	34	31.0	184	143	18	25.9	0.144
Zhang LW	2014	167	147	35	31.1	88	155	57	44.8	0.441
**rs231362**										
Been LF	2011	860	486	82	22.8	887	586	120	25.9	0.092
Ohshige T	2011	2310	460	18	8.9	1666	355	27	10.0	0.106
Iwata M	2012	606	102	5	7.9	619	123	6	9.0	0.968
Lu S	2012	402	86	3	9.4	285	105	9	15.4	0.854
Wang J	2012	132	128	40	34.7	30	53	17	43.5	0.434
Lin YD	2013	946	226	14	10.7	924	249	13	11.6	0.405
Riobello C	2016	39	94	47	52.2	114	262	125	51.1	0.299
Li YH (Uighur)	2018	160	107	17	24.8	24	53	22	49.0	0.479
Li YH (Chinese Han)	2018	228	64	4	12.2	161	47	0	11.3	0.066
**rs2283228**										
Unoki H (Japanese)	2008	1,379	1,592	449	36.4	426	590	217	41.5	0.606
Unoki H (Chinese)	2008	612	667	194	35.8	692	812	243	37.1	0.846
Unoki H (Caucasian)	2008	3,413	469	15	6.4	4,154	706	29	7.8	0.866
Chen Z	2010	21	29	7	37.7	135	162	44	36.7	0.671
Wei Q	2010	87	40	6	19.5	33	61	12	40.1	**0.042**
Saif-Ali R (Chinese)	2011	168	105	27	26.5	99	98	33	35.7	0.279
Saif-Ali R (Malay)	2011	141	72	21	24.4	73	83	21	35.3	0.725
Turki A	2013	812	77	2	4.5	519	53	3	5.1	0.203
Wang H	2013	1200	1093	240	31.1	1061	1246	336	36.3	0.314
Khan IA	2015	205	41	4	9.8	231	17	2	4.2	**0.014**
**rs151290**										
Yasuda K	2008	543	656	215	26.8	421	691	299	45.7	0.621
Wei Q	2010	43	64	26	43.6	17	60	29	55.7	0.131
Shi L	2011	87	61	23	31.3	108	144	36	37.5	0.258
Van JV	2011	2081	1220	153	22.1	2045	1258	204	23.8	0.567
Gao X	2012	47	107	46	49.8	41	109	50	52.3	0.192
Turki A	2012	465	343	71	27.6	312	209	34	25.0	0.899
Gao K	2016	294	358	71	34.6	287	364	107	38.1	0.624
Al-Shammari MS	2017	177	121	32	28.0	290	177	49	26.6	**0.005**
Huang Q	2018	197	247	60	36.4	181	234	80	39.8	0.764
**rs2074196**										
Yasuda K (Japanese)	2008	1674	2022	635	38.0	1335	2130	902	45.0	0.328

Yasuda K (Chinese)	2008	707	588	120	29.3	621	753	201	36.7	0.243
Yasuda K (Korean)	2008	316	363	76	34.1	212	309	109	41.8	0.843
Yasuda K (Caucasian)	2008	2542	225	4	4.2	3052	331	8	5.1	0.756
Hu C	2009	796	776	175	32.2	688	808	213	36.1	0.306

[a] MAF: minor allele frequency

WW, WM, and MM refers to CC, CT, and TT for **rs2237892;** WW, WM, and MM refers to AA, AC, and CC for **rs2237895;** WW, WM, and MM refers to CC, CT, and TT for **rs2237897;** WW, WM, and MM refers to GG, GA, and AA for **rs231362;** WW, WM, and MM refers to AA, AC, and CC for **rs2283228;** WW, WM, and MM refers to CC, CA, and AA for **rs151290;** WW, WM, and MM refers to GG, GT, and TT for **rs151290**

supplemental Table 3 Stratified analysis of *KCNQ1* gene seven polymorphisms on T2DM

Variables	N[a]	Cases/ Controls	Allelic comparison				Homozygote comparison				Heterozygote comparison				Dominant genetic model				Recessive genetic model			
			OR (95%CI)	P^b	I^2 (%)	P^c	OR (95%CI)	P^b	I^2(%)	P^c	OR (95%CI)	P^b	I^2(%)	P^c	OR (95%CI)	P^b	I^2(%)	P^c	OR (95%CI)	P^b	I^2(%)	P^c
**rs2237892**																						
Ethnicity																						
Asian	32	32636/28291	1.35 (1.28, 1.41)	**<0.001**	60.2	**<0.001**	1.91 (1.74, 2.10)	**<0.001**	45.7	**0.003**	1.45 (1.34, 1.57)	**<0.001**	29.8	**0.059**	1.67 (1.54, 1.81)	**<0.001**	36.0	**0.024**	1.39 (1.30, 1.48)	**<0.001**	60.2	**<0.001**
Caucasian	9	11242/13024	0.89 (0.59, 1.35)	0.583	95.3	**<0.001**	0.75 (0.35, 1.62)	0.459	74.5	**0.001**	0.77 (0.52, 1.14)	0.185	15.8	**0.309**	0.75 (0.40, 1.43)	0.387	63.0	**0.013**	0.86 (0.56, 1.34)	0.513	95.0	**<0.001**
Mixed	1	6869/8708	1.10 (1.00, 1.21)	0.054	-	-	1.63 (0.96, 2.79)	0.073	-	-	1.52 (0.88, 2.62)	0.132	-	-	1.62 (0.95, 2.77)	0.077	-	-	1.09 (0.99, 1.21)	0.097	-	-
HWE																						
Yes	34	35125/32338	1.32 (1.25, 1.38)	**<0.001**	59.5	**<0.001**	1.85 (1.66, 2.07)	**<0.001**	50.5	0.001	1.42 (1.29, 1.57)	**<0.001**	37.8	**0.017**	1.64 (1.49, 1.80)	**<0.001**	41.6	**0.008**	1.35 (1.26, 1.44)	**<0.001**	61.7	**<0.001**
No	8	15622/17685	0.99 (0.70, 1.42)	0.973	97.3	**<0.001**	1.15 (0.55, 2.38)	0.716	94.7	**<0.001**	1.27 (0.93, 1.74)	0.139	64.6	**0.006**	1.15 (0.64, 2.06)	0.651	91.7	**<0.001**	0.97 (0.66, 1.43)	0.876	96.8	**<0.001**
Control source																						
Population	18	40416/40381	1.30 (1.22, 1.37)	**<0.001**	71.2	**<0.001**	1.82 (1.6, 2.07)	**<0.001**	60.3	**0.001**	1.40 (1.24, 1.58)	**<0.001**	56.2	**0.002**	1.61 (1.43, 1.82)	**<0.001**	57.1	**0.002**	1.33 (1.24, 1.43)	**<0.001**	71.3	**<0.001**
Hospital	24	10331/9642	1.16 (0.97, 1.38)	0.111	92.2	**<0.001**	1.57 (1.15, 2.13)	**0.004**	85.5	**<0.001**	1.42 (1.22, 1.64)	**<0.001**	34.2	**0.056**	1.5 (1.18, 1.9)	**0.001**	77.9	**<0.001**	1.13 (0.91, 1.42)	0.269	91.3	**<0.001**
Score																						
<9	25	16490/17700	1.13 (0.95, 1.34)	0.178	92.3	**<0.001**	1.57 (1.13, 2.16)	**0.006**	85.7	**<0.001**	1.50 (1.26, 1.77)	**<0.001**	44.3	**0.011**	1.52 (1.18, 1.97)	**0.001**	78.9	**<0.001**	1.10 (0.90, 1.34)	0.375	91.3	**<0.001**
≥9	17	34257/32323	1.32 (1.26, 1.38)	**<0.001**	53.3	**0.005**	1.77 (1.58, 2.00)	**<0.001**	55.9	**0.003**	1.35 (1.21, 1.50)	**<0.001**	45.8	**0.024**	1.56 (1.40, 1.73)	**<0.001**	49.1	**0.014**	1.37 (1.30, 1.45)	**<0.001**	47.6	**0.015**
Year																						
<2015	27	39678/37037	1.30 (1.18, 1.43)	**<0.001**	91.6	**<0.001**	1.74 (1.45, 2.10)	**<0.001**	86.1	**<0.001**	1.38 (1.23, 1.55)	**<0.001**	61.4	**<0.001**	1.56 (1.34, 1.81)	**<0.001**	80.6	**<0.001**	1.33 (1.18, 1.49)	**<0.001**	90.6	**<0.001**

≥2015	15	11069/12986	1.11 (0.98, 1.25)	0.103	64.5	**<0.001**	1.65 (1.35, 2.02)	**<0.001**	20.8	0.227	1.53 (1.29, 1.81)	**<0.001**	0.0	0.993	1.61 (1.37, 1.89)	**<0.001**	0..0	0.720	1.07 (0.92, 1.24)	0.368	62.5	**0.001**
Number of participants																						
≤500	12	1723/1709	1.02 (0.81, 1.28)	0.875	69.7	**<0.001**	1.45 (0.96, 2.19)	0.076	51.6	**0.024**	1.63 (1.25, 2.13)	**<0.001**	0.0	0.459	1.54 (1.11, 2.13)	**0.010**	34.5	0.123	0.93 (0.70, 1.23)	0.618	66.4	**0.001**
>500	30	49024/48314	1.27 (1.17, 1.39)	**<0.001**	91.0	**<0.001**	1.73 (1.47, 2.04)	**<0.001**	84.1	**<0.001**	1.38 (1.25, 1.53)	**<0.001**	52.3	**0.001**	1.55 (1.36, 1.78)	**<0.001**	77.3	**<0.001**	1.31 (1.18, 1.45)	**<0.001**	90.1	**<0.001**
Influential analysis																						
Maximal	41	-/-	1.22 (1.13, 1.32)	**<0.001**	88.9	**<0.001**	1.66 (1.42, 1.94)	**<0.001**	80.2	**<0.001**	1.37 (1.26, 1.50)	**<0.001**	37.8	**0.010**	1.52 (1.35, 1.73)	**<0.001**	71.1	**<0.001**	1.22 (1.11, 1.34)	**<0.001**	87.8	**<0.001**
Minimal	41	-/-	1.30 (1.24, 1.37)	**<0.001**	67.6	**<0.001**	1.86 (1.68, 2.05)	**<0.001**	47.9	**0.001**	1.43 (1.31, 1.57)	**<0.001**	36.9	**0.002**	1.64 (1.51, 1.79)	**<0.001**	37.7	**0.010**	1.32 (1.24, 1.41)	**<0.001**	69.2	**<0.001**
**rs2237895**																						
Ethnicity																						
Asian	16	30077/24361	1.27 (1.22, 1.33)	**<0.001**	53.1	0.006	1.61 (1.48, 1.76)	**<0.001**	43.0	0.035	1.29 (1.24, 1.34)	**<0.001**	0.0	0.529	1.35 (1.28, 1.42)	**<0.001**	36.2	0.074	1.42 (1.33, 1.52)	**<0.001**	21.7	0.207
Caucasian	7	12050/13915	1.10 (1.02, 1.19)	**0.016**	72.6	0.001	0.75 (0.35, 1.62)	**0.029**	73.2	0.001	1.12 (1.03, 1.23)	**0.009**	47.0	0.079	1.15 (1.03, 1.27)	**0.010**	65.1	0.009	1.14 (1.01, 1.30)	**0.033**	61.7	0.016
HWE																						
Yes	21	41697/37663	1.21 (1.16, 1.27)	**<0.001**	73.3	**<0.001**	1.45 (1.32, 1.60)	**<0.001**	70.4	**<0.001**	1.23 (1.17, 1.29)	**<0.001**	51.3	0.004	1.28 (1.21, 1.35)	**<0.001**	66.3	**<0.001**	1.31 (1.22, 1.41)	**<0.001**	55.5	0.001
No	2	430/613	1.47 (0.72, 2.97)	0.288	89.9	0.002	1.8 (0.58, 5.58)	0.306	86.5	0.006	1.29 (0.83, 2.01)	0.257	40.1	0.196	1.54 (0.72, 3.29)	0.261	80.8	0.022	1.51 (0.62, 3.64)	0.364	83.0	0.015
Control source																						
Population	13	30497/27847	1.22 (1.15, 1.30)	**<0.001**	78.3	**<0.001**	1.48 (1.32, 1.67)	**<0.001**	74.6	**<0.001**	1.24 (1.16, 1.33)	**<0.001**	66.5	**<0.001**	1.29 (1.19, 1.39)	**<0.001**	74.9	**<0.001**	1.34 (1.23, 1.46)	**<0.001**	58.1	0.004
Hospital	10	11630/10429	1.20 (1.10, 1.31)	**<0.001**	68.9	**0.001**	1.40 (1.17, 1.67)	**<0.001**	67.1	**0.001**	1.22 (1.15, 1.30)	**<0.001**	0.0	0.657	1.26 (1.15, 1.38)	**<0.001**	45.4	0.058	1.26 (1.09, 1.46)	**0.002**	58.4	0.010
Score																						

<9	11	8568/8828	1.18 (1.09, 1.27)	**<0.001**	58.2	**0.008**	1.35 (1.15, 1.58)	**<0.001**	54.1	0.016	1.20 (1.12, 1.28)	**<0.001**	0.0	0.783	1.23 (1.14, 1.33)	**<0.001**	28.6	**0.073**	1.22 (1.07, 1.38)	**0.002**	42.1	0.069
≥9	12	33559/29448	1.24 (1.17, 1.31)	**<0.001**	79.9	**<0.001**	1.53 (1.36, 1.72)	**<0.001**	76.5	**<0.001**	1.25 (1.16, 1.34)	**<0.001**	68.9	**<0.001**	1.31 (1.21, 1.41)	**<0.001**	76.8	**<0.001**	1.37 (1.26, 1.49)	**<0.001**	60.5	0.003
Year																						
<2015	20	41517/37159	1.24 (1.19, 1.29)	**<0.001**	70.8	**<0.001**	1.52 (1.39, 1.66)	**<0.001**	66.1	**<0.001**	1.24 (1.19, 1.30)	**<0.001**	47.7	0.010	1.30 (1.23, 1.38)	**<0.001**	63.3	<0.001	1.35 (1.26, 1.44)	**<0.001**	48.9	0.007
≥2015	3	610/1117	0.89 (0.72, 1.10)	0.279	45.4	0.160	0.77 (0.50, 1.19)	0.238	43.7	0.169	0.97 (0.78, 1.21)	0.774	0.0	0.411	0.91 (0.69, 1.18)	0.460	31.5	0.232	0.83 (0.62, 1.11)	0.201	6.3	0.344
Number of participants																						
≤500	4	491/715	1.28 (0.86, 1.93)	0.229	77.8	0.004	1.54 (0.70, 3.4)	0.281	72.0	0.013	1.23 (0.90, 1.67)	0.188	18.2	0.300	1.33 (0.86, 2.06)	0.208	63.5	0.042	1.4 (0.75, 2.62)	0.288	61.7	0.050
>500	19	41636/37561	1.21 (1.16, 1.27)	**<0.001**	74.7	**<0.001**	1.45 (1.32, 1.59)	**<0.001**	72.0	**<0.001**	1.23 (1.17, 1.29)	**<0.001**	54.0	0.003	1.28 (1.21, 1.35)	**<0.001**	68.0	**<0.001**	1.31 (1.22, 1.41)	**<0.001**	58.1	0.001
Influential analysis																						
Maximal	22	-/-	1.20 (1.15, 1.26)	**<0.001**	70.1	**<0.001**	1.43 (1.30, 1.57)	**<0.001**	65.6	**<0.001**	1.22 (1.16, 1.28)	**<0.001**	44.4	0.014	1.27 (1.20, 1.34)	**<0.001**	62.2	**<0.001**	1.30 (1.20, 1.39)	**<0.001**	48.1	0.007
Minimal	22	-/-	1.23 (1.17, 1.28)	**<0.001**	66.5	**<0.001**	1.49 (1.35, 1.63)	**<0.001**	68.0	**<0.001**	1.26 (1.22, 1.30)	**<0.001**	6.3	0.377	1.30 (1.24, 1.37)	**<0.001**	48.6	0.006	1.34 (1.25, 1.43)	**<0.001**	50.8	**<0.001**
**rs2237897**																						
HWE																						
Yes	8	17014/16377	1.24 (1.04, 1.48)	**0.017**	95.0	**<0.001**	1.50 (1.02, 2.22)	**0.041**	94.1	**<0.001**	1.21 (0.96, 1.53)	0.111	86.0	**<0.001**	1.36 (0.99, 1.87)	0.058	93.4	**<0.001**	1.29 (1.07, 1.55)	**0.007**	90.8	**<0.001**
No	4	1794/2470	1.35 (1.10, 1.65)	**0.003**	52.6	**0.097**	1.58 (1.27, 1.96)	**<0.001**	0.0	0.596	1.08 (0.48, 2.46)	0.853	81.8	**0.004**	1.26 (0.74, 2.14)	0.388	61.3	**0.075**	1.66 (1.01, 2.72)	**0.046**	85.6	**<0.001**
Control source																						
Population	5	9280/9824	1.29 (1.01, 1.64)	**0.041**	96.6	**<0.001**	1.63 (1.00, 2.67)	0.050	95.7	**<0.001**	1.26 (0.96, 1.65)	0.092	88.7	**<0.001**	1.45 (0.99, 2.13)	0.054	95.1	**<0.001**	1.33 (1.02, 1.74)	**0.038**	93.7	**<0.001**
Hospital	7	9528/9023	1.26 (1.09, 1.47)	**0.002**	77.7	**<0.001**	1.35 (0.9, 2.03)	0.143	78.3	**<0.001**	1.08 (0.72, 1.6)	0.724	77.6	**<0.001**	1.21 (0.83, 1.78)	0.328	77.8	**<0.001**	1.39 (1.13, 1.72)	**0.002**	82.1	**<0.001**

Score																						
<9	6	3732/4438	1.26 (1.03, 1.53)	**0.022**	82.6	**<0.001**	1.29 (0.81, 2.03)	0.281	82.9	**<0.001**	1.02 (0.65, 1.60)	0.925	82.0	**<0.001**	1.15 (0.75, 1.77)	0.530	82.5	**<0.001**	1.43 (1.08, 1.89)	**0.014**	85.6	**<0.001**
≥9	6	15076/14409	1.29 (1.05, 1.58)	**0.015**	95.7	**<0.001**	1.68 (1.07, 2.64)	**0.025**	94.7	**<0.001**	1.27 (0.98, 1.64)	0.066	86.6	**<0.001**	1.48 (1.04, 2.12)	**0.031**	94.0	**<0.001**	1.33 (1.07, 1.65)	**0.011**	92.0	**<0.001**
Number of participants																						
≤500	3	361/735	1.46 (1.08, 1.97)	**0.014**	50.4	0.133	1.25 (0.72, 2.17)	0.435	0.0	0.628	0.89 (0.22, 3.52)	0.866	80.7	**0.023**	1.03 (0.45, 2.33)	0.951	52.0	0.149	1.91 (0.98, 3.72)	0.059	81.4	**0.005**
>500	9	18447/18112	1.24 (1.06, 1.44)	**0.007**	94.3	**<0.001**	1.52 (1.08, 2.14)	**0.016**	93.3	**<0.001**	1.25 (1.00, 1.55)	**0.045**	85.5	**<0.001**	1.39 (1.04, 1.84)	**0.025**	92.8	**<0.001**	1.28 (1.09, 1.50)	**0.003**	89.8	**<0.001**
Influential analysis																						
Maximal	11	-/-	1.24 (1.07, 1.42)	**0.003**	92.7	**<0.001**	1.38 (1.00, 1.91)	**0.049**	91.9	**<0.001**	1.15 (0.92, 1.45)	0.224	84.4	**<0.001**	1.28 (0.97, 1.68)	**0.083**	91.7	**<0.001**	1.30 (1.11, 1.52)	**0.001**	88.4	**<0.001**
Minimal	11	-/-	1.33 (1.22, 1.46)	**<0.001**	75.3	**<0.001**	1.71 (1.40, 2.08)	**<0.001**	72.0	**<0.001**	1.30 (1.09, 1.53)	**0.003**	61.6	**0.005**	1.49 (1.25, 1.76)	**<0.001**	66.4	**0.002**	1.44 (1.27, 1.63)	**<0.001**	78.9	**<0.001**
**rs2283228**																						
Ethnicity																						
Asian	7	8150/6477	1.34 (1.16, 1.55)	**<0.001**	81.3	**<0.001**	1.55 (1.24, 1.94)	**<0.001**	62.3	**0.014**	1.19 (1.07, 1.33)	**0.001**	0.0	0.708	1.33 (1.16, 1.52)	**<0.001**	26.9	0.223	1.48 (1.21, 1.81)	**<0.001**	82.6	**<0.001**
Caucasian	2	4788/5464	1.23 (1.10, 1.37)	**<0.001**	0.0	0.638	1.66 (0.92, 2.99)	0.093	0.0	0.687	1.36 (0.75, 2.48)	0.315	0.0	0.591	1.62 (0.90, 2.91)	0.111	0.0	0.671	1.23 (1.10 1.39)	**<0.001**	0.0	0.544
Mixed	1	250/250	0.40 (0.24, 0.68)	**0.001**	-	-	0.44 (0.08, 2.45)	0.351	-	-	1.21 (0.20, 7.22)	0.837	-	-	0.50 (0.09, 2.73)	0.421	-	-	0.38 (0.21, 0.66)	**0.001**	-	-
Score	7																					
<9	3	9850/8765	1.22 (0.90, 1.65)	0.199	87.2	**<0.001**	1.60 (1.02, 2.50)	**0.039**	60.1	**0.020**	1.06 (0.88, 1.28)	0.516	0.0	0.944	1.25 (0.98, 1.60)	0.078	14.9	0.316	1.31 (0.88, 1.95)	0.190	88.4	**<0.001**
≥9	7	3338/3426	1.25 (1.18, 1.32)	**<0.001**	0.0	0.940	1.58(1.38, 1.80)	**<0.001**	0.0	0.996	1.27 (1.11, 1.44)	**<0.001**	0.0	0.904	1.41 (1.25, 1.59)	**<0.001**	0.0	0.950	1.29 (1.21, 1.39)	**<0.001**	0.0	0.675
Number of participants																						

≤500	4	674/874	1.18 (0.58, 2.38)	0.645	91.8	**<0.001**	1.64 (0.71, 3.80)	0.245	63.7	**0.041**	1.03 (0.65, 1.64)	0.895	0.0	0.916	1.35 (0.83, 2.20)	0.224	12.6	0.330	1.33 (0.50, 3.52)	0.568	93.0	**<0.001**
>500	6	12514/11317	1.22 (1.13, 1.32)	**<0.001**	56.0	**0.045**	1.47 (1.24, 1.76)	**<0.001**	45.7	0.101	1.20 (1.08, 1.34)	**0.001**	0.0	0.650	1.33 (1.16, 1.51)	**<0.001**	24.9	0.247	1.26 (1.15, 1.37)	**<0.001**	43.8	0.113
Influential analysis																						
Maximal	9	-/-	1.19 (1.06, 1.34)	**0.003**	76.5	**<0.001**	1.46 (1.24, 1.73)	**<0.001**	35.9	0.131	1.20 (1.07, 1.33)	**0.001**	0.0	0.873	1.31 (1.18, 1.46)	**<0.001**	2.2	0.416	1.22 (1.05, 1.42)	**0.008**	77.0	**<0.001**
Minimal	9	-/-	1.30 (1.16, 1.45)	**<0.001**	75.3	**<0.001**	1.55 (1.27, 1.89)	**<0.001**	50.6	**0.039**	1.20 (1.08, 1.34)	**0.001**	0.0	0.909	1.33 (1.18, 1.49)	**<0.001**	10.9	0.342	1.38 (1.19, 1.61)	**<0.001**	77.5	**<0.001**
**rs231362**																						
Ethnicity																						
Asian	7	6058/4532	1.19 (0.73, 1.94)	0.492	95.9	**<0.001**	1.63 (0.71, 3.74)	0.250	80.0	**<0.001**	1.35 (0.88, 2.09)	0.174	32.2	0.182	1.42 (0.75, 2.70)	0.280	68.9	**0.004**	1.28 (0.74, 2.21)	0.386	95.7	**<0.001**
Caucasian	2	1608/2094	1.09 (0.89, 1.35)	0.393	60.2	0.113	1.19 (0.78, 1.82)	0.424	56.2	0.131	1.11 (0.87, 1.42)	0.389	0.0	0.356	1.15 (0.82, 1.62)	0.422	50.4	0.156	1.15 (0.95, 1.39)	0.155	20.4	0.262
Control source																						
Population	5	3374/3587	1.26 (0.95, 1.67)	0.113	87.7	**<0.001**	1.53 (0.71, 3.29)	0.282	85.6	**<0.001**	1.18 (0.80, 1.74)	0.408	52.5	**0.077**	1.36 (0.78, 2.39)	0.279	78.0	**0.001**	1.31 (0.96, 1.79)	0.087	83.3	**<0.001**
Hospital	4	4292/3039	1.04 (0.49, 2.19)	0.923	96.9	**<0.001**	1.44 (0.68, 3.05)	0.338	58.2	**0.066**	1.30 (0.83, 2.04)	0.249	0.0	0.574	1.26 (0.71, 2.25)	0.436	36.0	0.196	1.08 (0.46, 2.55)	0.858	97.0	**<0.001**
Score																						
<9	3	1075/598	1.94 (1.32, 2.87)	0.563	77.7	**0.011**	4.03 (1.41, 11.51)	**0.009**	76.4	**0.014**	1.74 (0.88, 3.45)	0.111	49.7	0.137	2.68 (1.10, 6.52)	0.517	72.1	**0.028**	2.31 (1.43, 3.73)	**0.001**	72.5	**0.026**
≥9	6	6591/6028	0.90 (0.63, 1.29)	**0.001**	94.7	**<0.001**	1.04 (0.76, 1.44)	0.798	28.1	0.224	1.11 (0.89, 1.38)	0.377	0.0	0.526	1.08 (0.85, 1.37)	**0.030**	8.5	0.362	0.90 (0.59, 1.38)	0.624	94.9	**<0.001**
Number of participants																						
≤500	2	584/199	2.05 (1.04, 4.06)	**0.039**	88.1	**0.004**	3.98 (0.89, 17.81)	0.071	88.2	**0.004**	1.62 (0.65, 4.04)	0.303	72.2	**0.058**	2.42 (0.74, 7.97)	0.145	85.1	**0.010**	2.71 (1.25, 5.86)	**0.012**	79.0	**0.029**
>500	7	7082/6427	0.99 (0.70, 1.39)	0.941	94.6	**<0.001**	1.10 (0.75, 1.61)	0.617	43.5	0.101	1.13 (0.91, 1.40)	0.277	0.0	0.481	1.10 (0.80, 1.49)	0.565	31.1	0.191	0.99 (0.66, 1.48)	0.967	94.7	**<0.001**

Influential analysis																						
Maximal	8	-/-	1.03 (0.75, 1.42)	0.839	94.0	**<0.001**	1.19 (0.85, 1.68)	0.318	42.0	**0.098**	1.12 (0.91, 1.37)	0.291	0.0	0.590	1.13 (0.88, 1.46)	0.341	21.5	0.258	1.06 (0.73, 1.55)	0.755	94.2	**<0.001**
Minimal	8	-/-	1.32 (1.07, 1.61)	**0.008**	82.7	**<0.001**	1.70 (0.98, 2.93)	0.058	77.2	**<0.001**	1.31 (0.96, 1.77)	0.088	23.0	0.246	1.44 (0.96, 2.18)	0.081	66.1	**0.004**	1.39 (1.11, 1.74)	**0.005**	78.2	**<0.001**
**rs151290**																						
Ethnicity																						
Asian	6	3145/3258	1.26 (1.15, 1.37)	**<0.001**	20.9	0.276	1.64 (1.41, 1.90)	**<0.001**	0.0	0.458	1.26 (1.06, 1.50)	**0.009**	19.9	0.283	1.42 (1.24, 1.62)	**<0.001**	0.0	0.566	1.35 (1.13, 1.62)	**0.001**	57.0	**0.040**
Caucasian	3	4663/4578	0.98 (0.83, 1.15)	0.811	70.6	**0.033**	1.00 (0.66, 1.52)	0.994	73.6	**0.023**	1.07 (0.79, 1.46)	0.662	50.2	0.134	1.03 (0.71, 1.49)	0.894	68.6	**0.041**	0.98 (0.84, 1.15)	0.806	52.5	0.122
Control source																						
Population	4	6095/6171	1.19 (1.07, 1.32)	**0.001**	67.8	**0.025**	1.55 (1.34, 1.78)	**<0.001**	8.6	0.350	1.34 (1.18, 1.53)	**<0.001**	0.0	0.912	1.44 (1.27, 1.62)	**<0.001**	0.0	0.861	1.19 (1.02, 1.40)	**0.031**	72.0	**0.013**
Hospital	5	1713/1665	1.10 (0.90, 1.36)	0.356	70.4	**0.009**	1.16 (0.78, 1.72)	0.475	61.1	**0.036**	0.92 (0.73, 1.17)	0.506	0.0	0.581	0.99 (0.79, 1.24)	0.909	5.0	0.378	1.24 (0.88, 1.73)	0.216	77.7	**0.001**
Score																						
<9	5	1713/1665	1.10 (0.90, 1.36)	0.356	70.4	**0.009**	1.16 (0.78, 1.72)	0.475	61.1	**0.036**	0.92 (0.73, 1.17)	0.506	0.0	0.581	0.99 (0.79, 1.24)	0.909	5.0	0.378	1.24 (0.88, 1.73)	0.216	77.7	**0.001**
≥9	4	6095/6171	1.19 (1.07, 1.32)	**0.001**	67.8	**0.025**	1.55 (1.34, 1.78)	**<0.001**	8.6	0.350	1.34 (1.18, 1.53)	**<0.001**	0.0	0.912	1.44 (1.27, 1.62)	**<0.001**	0.0	0.861	1.19 (1.02, 1.40)	**0.031**	72.0	**0.013**
Year																						
<2015	6	6251/6067	1.17 (1.01, 1.37)	**0.038**	79.4	**<0.001**	1.36 (1.01, 1.84)	**0.042**	71.7	**0.003**	1.11 (0.91, 1.36)	0.288	42.4	0.122	1.20 (0.97, 1.49)	0.092	54.9	**0.050**	1.28 (1.03, 1.61)	**0.028**	81.5	**<0.001**
≥2015	3	1557/1769	1.10 (0.97, 1.25)	0.150	33.8	**0.221**	1.33 (1.01, 1.76)	**0.045**	32.2	0.229	1.36 (1.09, 1.70)	**0.007**	0.0	0.514	1.34 (1.06, 1.70)	**0.014**	16.2	0.303	1.06 (0.92, 1.22)	0.413	0.0	0.415
Number of participants																						
≤500	3	504/594	1.30 (1.06, 1.60)	**0.013**	27.3	0.253	1.55 (0.96, 2.49)	0.071	39.3	0.192	0.96 (0.69, 1.33)	0.790	3.4	0.355	1.15 (0.84, 1.56)	0.384	0.0	0.458	1.66 (1.15, 2.41)	**0.007**	43.2	0.172

>500	6	7304/7242	1.10 (0.97, 1.24)	0.128	78.3	**<0.001**	1.30 (1.01, 1.65)	**0.039**	71.3	**0.004**	1.26 (1.09, 1.46)	**0.001**	21.1	0.275	1.28 (1.06, 1.53)	**0.010**	55.2	**0.048**	1.09 (0.94, 1.27)	0.239	74.1	**0.002**
Sensitivity analysis																						
Maximal	8	-/-	1.10 (1.00, 1.22)	0.057	56.0	**0.026**	1.27 (1.02, 1.58)	**0.031**	51.6	**0.043**	1.15 (0.97, 1.38)	**0.115**	32.5	0.169	1.20 (1.01, 1.43)	**0.038**	35.8	0.143	1.12 (0.97, 1.30)	0.114	61.6	**0.011**
Minimal	8	-/-	1.18 (1.07, 1.30)	**0.001**	59.9	**0.015**	1.47 (1.25, 1.73)	**<0.001**	33.1	0.164	1.27 (1.13, 1.43)	**<0.001**	0.0	0.445	1.28 (1.10, 1.51)	**0.002**	41.5	0.521	1.23 (1.05, 1.44)	**0.008**	70.4	**0.001**
**rs2074196**																						
Influential analysis																						
Maximal	4	-/-	1.29 (1.20, 1.38)	**<0.001**	41.0	0.166	1.70 (1.49, 1.94)	**<0.001**	25.1	0.261	1.31 (1.19, 1.44)	**<0.001**	0.0	0.745	1.48 (1.35, 1.62)	**<0.001**	0.0	0.513	1.34 (1.23, 1.46)	**<0.001**	30.6	0.229
Minimal	4	-/-	1.35 (1.28, 1.41)	**<0.001**	0.0	0.612	1.83 (1.65, 2.04)	**<0.001**	0.0	0.775	1.37 (1.24, 1.52)	**<0.001**	0.0	0.634	1.56 (1.41, 1.71)	**<0.001**	0.0	0.672	1.40 (1.29, 1.53)	**<0.001**	37.2	0.189

CI: Confidence interval

[a] Number of comparisons

[b] *P*-value of Z-test for significant test

[c] *P*-value of Q-test for between-study heterogeneity test

supplemental Table 4 The meta-regression analysis of *KCNQ1* gene homozygous on T2DM

	Coefficient	Standard error	t	*P* value	95% CI of intercept
**rs2237892**					
N	-0.0000149	0.0000137	-1.09	0.282	(-0.0000426, 0.0000128)
Control source	0.0145201	0.1043601	0.14	0.890	(-0.1967458, 0.225786)
Publication year	-0.0061387	0.0165013	-0.37	0.712	(-0.0395439, 0.0272666)
Age	0.0010279	0.0087	0.12	0.907	(-0.0166725, 0.0187283)
Ethnicity	-0.2705131	0.1275828	-2.12	**0.041**	(-0.528791, -0.0122353)
Score	-0.0216028	0.0254766	-0.85	0.402	(-0.0731775, 0.0299719)
HWE	0.1810266	0.1870884	0.97	0.339	(-0.1977141, 0.5597673)
**rs2237895**					
N	0.0000209	0.0000152	1.37	0.185	(-0.0000108, 0.0000525)
Control source	-0.0552132	0.118778	-0.46	0.647	(-0.3022255, 0.1917991)
Publication year	-0.0436679	0.0203493	-2.15	**0.044**	(-0.0859865, -0.0013493)
Age	0.0002093	0.0097558	0.02	0.983	(-0.0204721, -0.0204721)
Ethnicity	-0.2668295	0.0917712	-2.91	**0.008**	(-0.4576782, -0.0759809)
Score	0.0240586	0.0311911	0.77	0.449	(-0.040807, 0.0889241)
HWE	-0.0246308	0.2712333	-0.09	0.929	(-0.5886912, 0.5394296)
**rs2237897**					
N	0.0000461	0.0000784	0.59	0.571	(-0.0001312, 0.0002235)
Control source	-0.195589	0.3597079	-0.54	0.600	(-1.009305, 0.6181268)
Publication year	-0.0944947	0.0705882	-1.34	0.214	(-0.2541763, 0.065187)
Age	-0.0073832	0.0408886	-0.18	0.863	(-0.107434, 0.0926675)
Ethnicity	0.6293816	0.8635118	0.73	0.485	(-1.324018, 2.582781)
Score	0.0482924	0.0813932	0.59	0.568	(-0.1358318, 0.2324166)
HWE	0.0463805	0.4222846	0.11	0.915	(-0.9088937, 1.001655)
**rs2283228**					
N	-0.0000209	0.0000513	-0.41	0.694	(-0.0001392, 0.0000973)
Control source	-0.0301486	0.3143583	-0.10	0.926	(-0.7550602, 0.694763)
Publication year	0.0246078	0.0554848	0.44	0.669	(-0.1033404, 0.1525561)
Age	0.0483061	0.042533	1.14	0.299	(-0.0557706, 0.1523829)
Ethnicity	-0.2017557	0.3257576	-0.62	0.553	(-0.9529542, 0.5494428)
Score	-0.020264	0.1070768	-0.19	0.855	(-0.2671834, 0.2266555)
HWE	-0.6400612	0.3358988	-1.91	0.093	(-1.414645, 0.1345229)
**rs231362**					
N	-0.0002959	0.000208	-1.42	0.198	(-0.0007877, 0.0001959)
Control source	-0.0156146	0.6932902	-0.02	0.983	(-1.654985, 1.623756)
Publication year	0.1013156	0.127708	0.79	0.454	(-0.2006658, 0.403297)
Age	-0.1128555	0.0575861	-1.96	0.107	(-0.2608853, 0.0351743)
Ethnicity	-0.3716854	0.7474098	-0.50	0.634	(-2.139029, 1.395658)

Score	-0.1932001	0.1591486	-1.21	0.264	(-0.5695267, 0.1831266)
HWE	1.381958	1.01491	1.36	0.216	(-1.017922, 3.781838)
**rs151290**					
N	9.75e-06	0.0000566	0.17	0.868	(-0.0001241, 0.0001436)
Control source	-0.3452573	0.2034566	-1.70	0.134	(-0.8263557, 0.1358412)
Publication year	-0.0292026	0.0355577	-0.82	0.439	(-0.1132832, 0.0548781)
Age	0.0292181	0.0247505	1.18	0.282	(-0.0313442, 0.0897804)
Ethnicity	-0.4198043	0.184458	-2.28	**0.057**	(-0.8559781, 0.0163696)
Score	0.0343289	0.0376308	0.91	0.392	(-0.0546538, 0.1233116)
HWE	-0.1654653	0.4434893	-0.37	0.720	(-1.214151, 0.8832203)

N total number of participants

supplementary Table 5 Publication bias tests (Egger's funnel plot for publication bias test) for *KCNQ1* gene seven polymorphisms

Genetic type	Coefficient	Standard error	t	*P* value	95%CI of intercept
**rs2237892**					
Allelic contrast	-1.078	0.788	-1.37	0.179	-2.671, 0.516
Homozygote comparison	-0.521	0.590	-0.88	0.382	-1.715, 0.672
Heterozygote comparison	0.120	0.353	0.34	0.737	-0.595, 0.834
Dominant genetic model	-0.223	0.496	-0.45	0.656	-1.226, 0.781
Recessive genetic model	-1.119	0.764	-1.47	0.151	-2.662, 0.424
**rs2237895**					
Allelic contrast	-0.527	0.849	-0.62	0.541	-2.293, 1.239
Homozygote comparison	-0.611	0.784	-0.78	0.445	-2.242, 1.020
Heterozygote comparison	-0.085	0.612	-0.14	0.891	-1.358, 1.188
Dominant genetic model	-0.355	0.750	-0.47	0.641	-1.914, 1.204
Recessive genetic model	-0.603	0.636	-0.95	0.354	-1.925, 0.719
**rs2237897**					
Allelic contrast	11.800	8.888	1.33	0.214	-8.004, 31.605
Homozygote comparison	8.520	6.604	1.29	0.229	-6.42, 23.460
Heterozygote comparison	0.806	1.441	0.56	0.590	-2.453, 4.065
Dominant genetic model	6.149	3.512	1.75	0.114	-1.795, 14.093
Recessive genetic model	6.847	6.675	1.03	0.329	-8.025, 21.719
**rs2283228**					
Allelic contrast	0.227	1.431	0.16	0.878	-3.073, 3.528
Homozygote comparison	0.435	0.751	0.58	0.579	-1.298, 2.167
Heterozygote comparison	0.038	0.367	0.10	0.921	-0.809, 0.884
Dominant genetic model	0.259	0.557	0.46	0.655	-1.026, 1.544
Recessive genetic model	0.374	1.529	0.24	0.813	-3.151, 3.899
**rs231362**					
Allelic contrast	2.218	4.119	0.54	0.607	-7.523, 11.959
Homozygote comparison	0.126	1.460	0.09	0.933	-3.325, 3.578
Heterozygote comparison	0.041	0.807	0.05	0.961	-1.868, 1.950
Dominant genetic model	0.029	1.170	0.03	0.981	-2.736, 2.795
Recessive genetic model	3.167	3.587	0.88	0.407	-5.315, 11.649
**rs151290**					
Allelic contrast	0.077	1.497	0.05	0.960	-3.463, 3.618
Homozygote comparison	-1.066	1.391	-0.77	0.468	-4.354, 2.222
Heterozygote comparison	-1.820	0.856	-2.13	0.071	-3.843, 0.204
Dominant genetic model	-1.875	0.996	-1.88	0.102	-4.231, 0.481
Recessive genetic model	1.083	1.336	0.81	0.444	-2.076, 4.242
**rs2074196**					
Allelic contrast	0.502	1.791	0.28	0.798	-5.199, 6.202
Homozygote comparison	-0.131	1.199	-0.11	0.920	-3.947, 3.686
Heterozygote comparison	0.614	1.688	0.36	0.740	-4.758, 5.987
Dominant genetic model	1.085	2.107	0.51	0.642	-5.621, 7.791
Recessive genetic model	-0.365	1.008	-0.36	0.742	-3.573, 2.844
